# Dynamic Supercooled Crystal–Gels for Ultrafast, Reversible Mechanical Adaptative Materials

**DOI:** 10.1002/advs.78566

**Published:** 2026-11-03

**Authors:** Yuesong Lv, Hao Feng, Zhangqin Yang, Jianping Yin, Hanfei Li, Bo Zhou, Yuyang Meng, Yuntong Su, Xinxing Zhang, Xibo Pei

**Affiliations:** ^1^ National Key Laboratory of Advanced Polymer Materials Polymer Research Institute Sichuan University Chengdu China; ^2^ State Key Laboratory of Oral Diseases National Clinical Research Center for Oral Diseases Department of Prosthodontics West China Hospital of Stomatology Sichuan University Chengdu China; ^3^ Shaanxi Key Laboratory of Impact Dynamics and Its Engineering Application School of Aeronautics Northwestern Polytechnical University Xi'an China

**Keywords:** dynamic crystallization network, mechanical‐adaptive materials, seconds‐scale release microneedles, stimulus‐stiffening, supercooled crystal‐gels

## Abstract

Endowing synthetic materials with adaptability and responsiveness to dynamically optimize their states represents a premier frontier in biomimetic science. However, achieving life‐like, rapid yet reversible adaptation under benign triggers remains hindered by high reconfiguration energy or delayed structural recovery. In this work, we present dynamic supercooled crystal‐gels that achieve ultrafast, reversible stiffening without requiring continuous energy input. Inspired by the tactile‐triggered stiffening of starfish tissues, it utilizes non‐equilibrium energy of internal supercooled liquid to realize templated crystallization upon a benign tactile stimulus trigger. Meanwhile, the crystalline structure is reversible under appropriate temperature and humidity perturbations. This synchronously achieves a 2596‐fold adjustment in compressive modulus, circumventing the conventional trade‐off between energy demands for mechanical reinforcement and structural stability. As a functional validation, microneedles based on supercooled crystal‐gels fulfill the divergent requirements for skin adaptation and seconds‐scale drug release through their mechanical adaptation, achieving rapid cumulative drug release of ≈37.76% at 10 s and 88.12% within 5 min. This work delineates a strategy for endowing materials with autonomous mechanical adaptation and opens new opportunities for life‐like functional evolution in soft robotics and bio‐integrated electronics.

## Graphical abstract

A dynamic supercooled crystal–gel mimics starfish tissue by storing non‐equilibrium energy in a metastable liquid. A gentle touch triggers templated crystallization, producing a 2596‐fold stiffness increase with negligible volume change. The lattice disintegrates reversibly under humidity or warmth. Developed Microneedles exploit this biomechanical logic for skin penetration and seconds‐scale drug release.

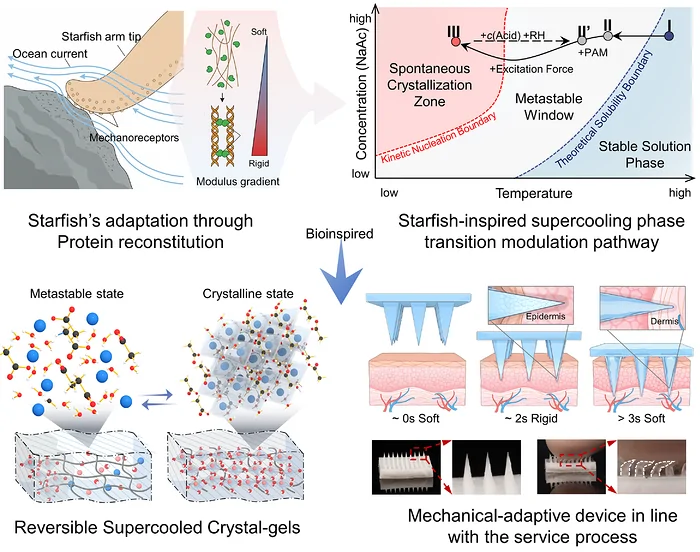

## 1 Introduction

Nature often achieves the synergy between adaptive responsiveness and functional performance through tissue‐level responses to environmental stimuli, as exemplified by starfish, which exhibit rapid and reversible mechanical adaptation via mechanoreceptor‐regulated, variably cross‐linked collagenous tissues [1, 2]. Inspired by these biological principles, biomimetic materials based on dynamic chemical networks or phase transitions have been developed to convert environmental adaptability into functional performance, advancing applications in bio‐integrated electronics and soft robotics under complex physiological conditions [3, 4, 5, 6, 7, 8]. However, as these frontier fields evolve, they increasingly demand “living materials” that transcend simple passive responsiveness, requiring instead an autonomous, life‐like logic that can rapidly adapt its mechanical state precisely when and where the application dictates [9, 10, 11].

Endeavors have been dedicated to engineering reconfigurable materials in pursuit of such life‐like environmental adaptability, such as stimuli‐responsive hydrogels [12, 13], shape‐memory polymers [14, 15], and magnetically responsive elastomers [16, 17, 18]. These paradigms predominantly rely on external triggers to induce rapid molecular realignment, network shuffling, or macroscopic swelling [19]. However, a fundamental challenge persists that the realization of ultrafast responsiveness typically occurs at the severe demand of extreme conditions such as extreme light, heat, electricity, etc [20, 21, 22]. Most adaptive responses without additional stimulus exhibit a “negative‐gain” behavior, wherein rapid structural actuations or phase transitions inherently provoke polymer chain relaxation, culminating in macroscopic softening or yielding [23]. Consequently, while current materials can efficiently execute fast and reversible functional alterations, they fail to spontaneously optimize their mechanical state like what living organisms do during adaptation [24].

From a physical chemistry perspective, driving substantial and rapid mechanical reinforcement typically necessitates surmounting significant thermodynamic or kinetic barriers to firmly maintain the newly formed rigid domains [25, 26]. For instance, recent state‐of‐the‐art composites based on vibration‐induced crosslinking and thermal‐hyperhardening hydrogels achieve massive positive modulus amplifications, which depend on continuous mechanical agitation or elevated thermal driving forces [27, 28]. Conversely, even when sophisticated phase transitions are harnessed to encode morphological logic, such as shape memory polymers with programmable recovery onsets, materials under benign ambient triggers (e.g., moisture, physiological heat) frequently suffer a monotonic decline in strength due to plasticization or topological migrations [6, 29, 30, 31]. This leaves a paradox between overcoming the thermodynamic barrier to reinforcement and maintaining high‐fidelity structural locking [32, 33]. Therefore, we suppose that the key to achieving autonomous life‐like “positive‐gain” mechanical adaptation lies in shifting the focus from stimulus magnitude to the kinetic modulation of a pre‐stored, non‐equilibrium energy landscape.

Herein, we report an ultrafast, reversible, positive‐gain mechanical adaptive material based on dynamic supercooled crystal‐gels (SCGs). By in situ stabilizing the metastable supercooled liquid within a polyacrylamide (PAM) gel scaffold, we elevate its critical nucleation barrier to obtain the pre‐stored energy landscape. Following the functional logic of tactile‐triggered stiffening in starfish, a benign mechanical stimulus triggers the release of stored thermodynamic energy and rapidly initiates crystallization. Crucially, the PAM network functions as a templating matrix for heterogeneous nucleation to guide molecular clusters into ordered crystalline bundles, successfully translating micro‐scale phase reconfiguration into seconds‐scale, reversible modulus amplifications. As a proof of concept, crystal‐gels were fabricated into drug‐loaded microneedles. Their “soft‐stiff‐soft” mechanical logic fulfills the divergent modulus requirements for skin penetration and drug release, validated in porcine models for emergency administration. This work offers a strategy for achieving autonomous mechanically adaptative materials at room‐temperature and delineates a new frontier for next‐generation living materials.

## 2 Results and Discussion

### 2.1 Bioinspired Materials Design of Supercooled Crystal‐Gels

Traditional reinforcement strategies like strain‐induced crystallization, shear‐thickening, or thermally‐driven phase separation primarily present inherent limitations, either necessitating persistent energy dissipation or relying on irreversible macroscopic deformation [34, 35, 36]. In contrast, the soft‐to‐stiff adaptation of starfish mutable collagenous tissue illustrates a high‐gain mechanical‐regulation strategy (Figure 1a). In this biological system, low‐intensity environmental cues are converted into neural and chemical regulation of interfibrillar cohesion within the collagenous network, thereby enhancing load transfer and tissue stiffness [37, 38, 39]. Our design emulates the same functional regulatory logic, in which a small local input induces internal structural reorganization and produces a large mechanical output. In SCGs, this logic is implemented through a minute mechanical perturbation that initiates crystallization powered by the pre‐stored nonequilibrium free energy. On this basis, we propose a bioinspired kinetic‐management strategy based on a functional analogy to starfish tissue stiffening.

**FIGURE 1:**
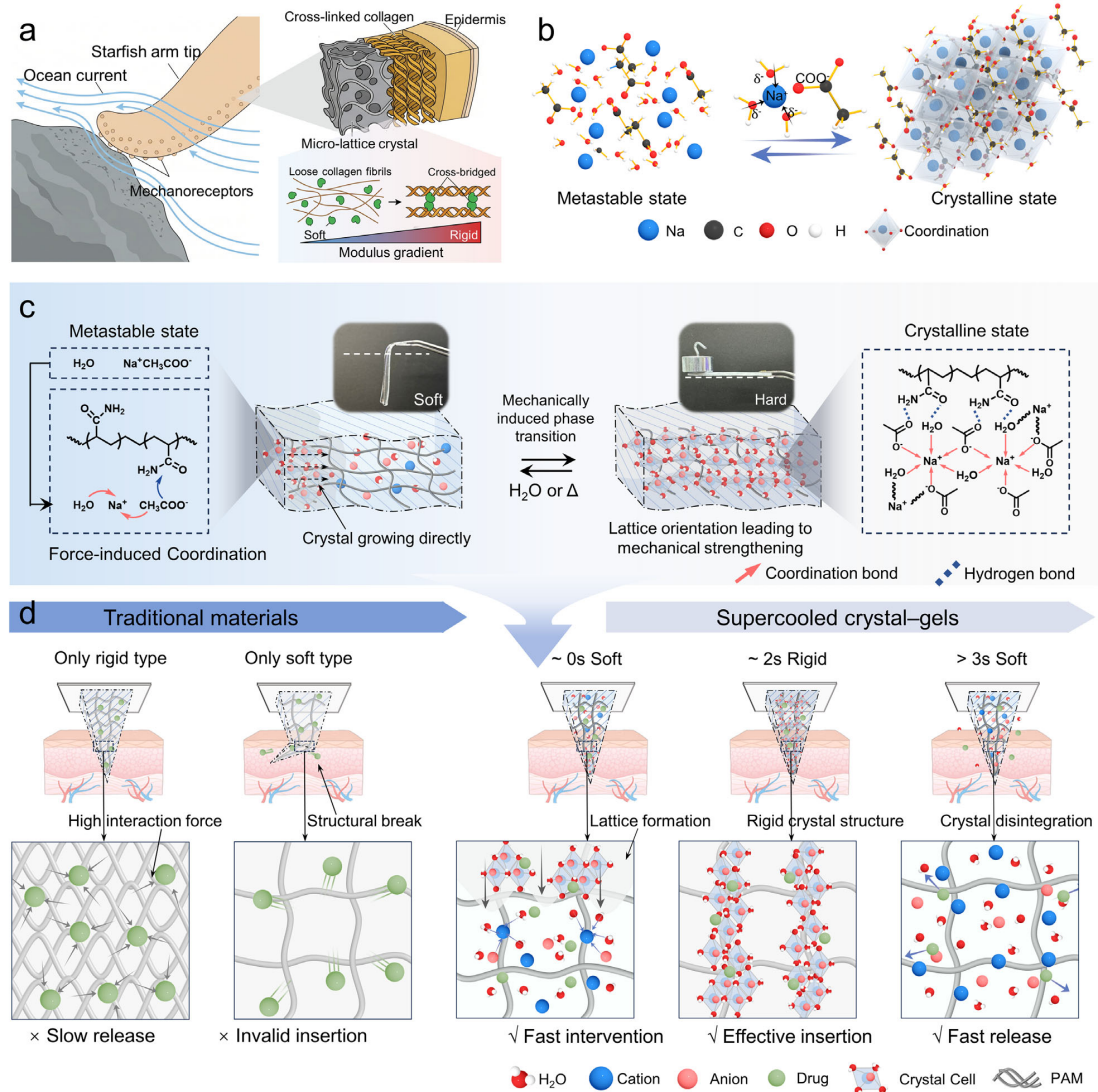
Bioinspired design strategy and conceptual application of the SCGs. (a) Schematic illustration of the tactile‐triggered soft‐to‐stiff adaptation of starfish tube feet. External mechanical cues are transduced into biological signals that regulate interfibrillar cohesion within the mutable collagenous tissue, thereby enhancing load transfer through the collagenous network and increasing tissue stiffness. (b) Molecular schematic of the crystallization in supercooled sodium acetate crystals. (c) Evolution of dynamic interactions during the ordered, mechanoresponsive transition steered by the PAM templating. (d) Optimized penetration and drug‐release logic in microneedle applications, enabled by the mechanical adaptation.

Rather than relying on energy‐intensive physical processing, our design directly harnesses the pre‐stored, non‐equilibrium energy landscape of supercooled sodium acetate (Figure 1b). By spatially confining this metastable phase within a polyacrylamide (PAM) framework, the critical nucleation barrier is substantially elevated via electrostatic mismatch, stabilizing a highly sensitive, fluidic ground state. Upon minute mechanical perturbations, the PAM scaffold functions as a templating matrix, steering the stochastic free growth into an ordered, directional lattice evolution (Figure 1c). This templated confinement inherently decouples triggering sensitivity from reinforcement energy costs, driving an ultrafast, dynamic, “positive‐gain” modulus amplification. Translating this physical logic into functional devices, SCGs were conceptualized into adaptive microneedles (Figure 1d), where their autonomous “soft‐stiff‐soft” reconfiguration precisely fulfills the divergent biomechanical demands for rigid tissue penetration and subsequent compliant drug release in physiological environments.

### 2.2 Reversible Supercooled Phase Transformation

SCGs are designed to harness the pre‐stored enthalpy and spontaneous transition potential of supercooled states. By mapping the phase stability of the sodium acetate (NaAc) system, we identified a metastable window (Zone II, Figure 2a) where transition kinetics are highly responsive to environmental variables (Figure S1). Introducing a polyacrylamide (PAM) framework alters this thermodynamic landscape. The weak interfacial interaction between the PAM matrix and solvated clusters raises the critical nucleation barrier, expanding the metastable regime into a stabilized “Zone II’” (Figure 2b). This kinetic entrapment preserves a sensitive, high‐entropy base state while preventing spontaneous crystallization. Upon mechanical stimulation, localized kinetic and electrochemical perturbations overcome the barrier at the point of contact to trigger initial nucleation. Crucially, this localized event acts as an unlocking mechanism [40]. The instantaneous release of pre‐stored latent heat locally diminishes the kinetic threshold for adjacent solvated clusters, driving a self‐sustaining crystallization wavefront. Unlike the disordered 3D growth in bulk systems, the PAM network acts as a scaffold, guiding the crystallization front along the polymer chains.

**FIGURE 2:**
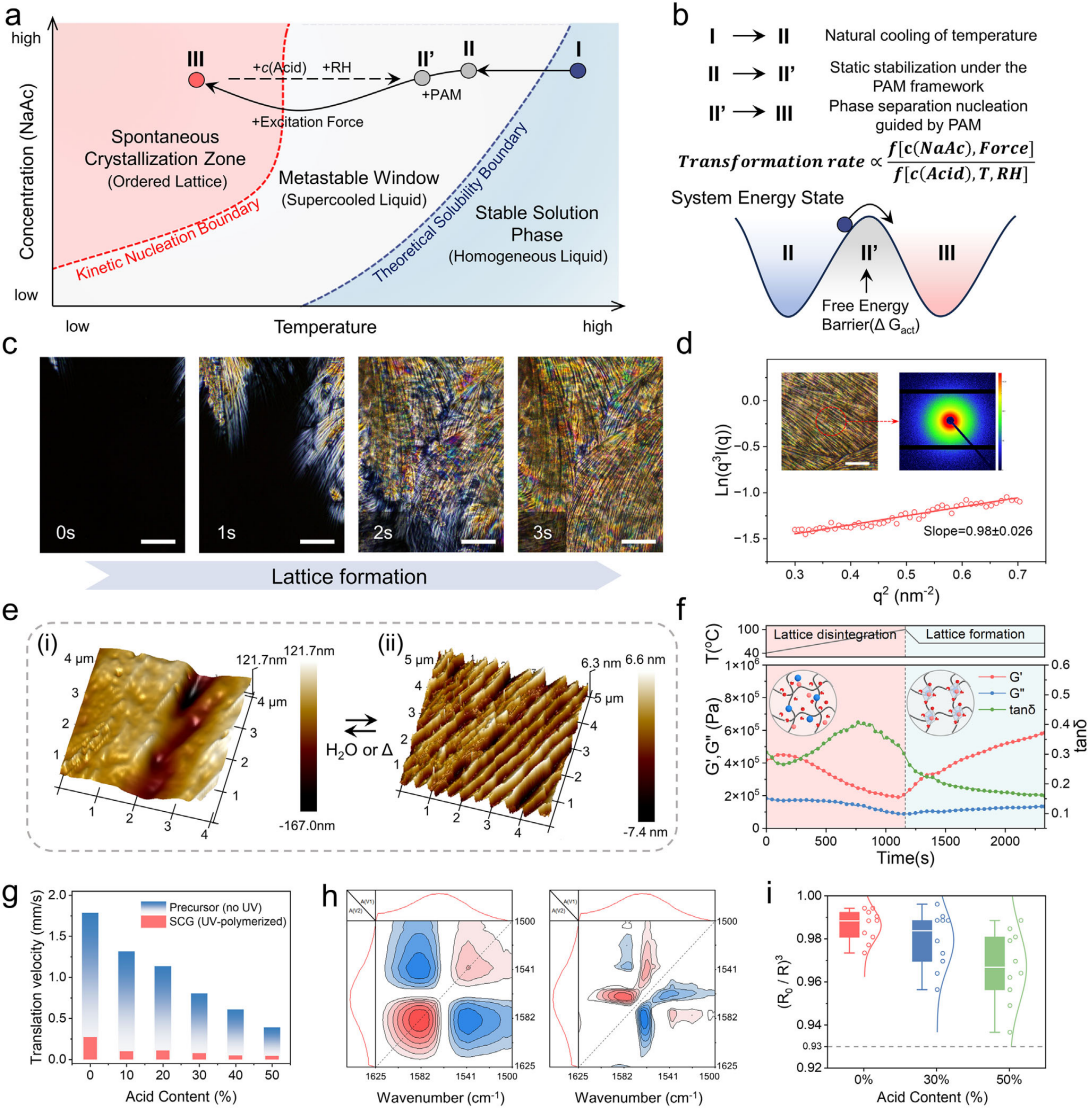
Dynamic crystallization processes and kinetics of SCGs. (a) Global phase stability map of SCGs illustrating the Stable Solution Phase (Zone I), Metastable Window (Zone II), and Spontaneous Crystallization Zone (Zone III) as a function of temperature and concentration (NaAc). (b) Detailed mechanisms of the transition and its free energy landscape, demonstrating the transition from a metastable supercooled state to a crystalline state. (c) In situ polarized optical microscopy images capturing the crystal growth behavior. Scale bar, 100 µm. (d) SAXS plots of *ln(q^3^I(q))* vs. *q^2^* of SCGs. Insets display the characterized oriented crystalline prism and its corresponding 2D SAXS pattern. Scale bar, 50 µm. (e) Representative 3D surface topographies of (i) amorphous and (ii) latticed SCGs obtained via AFM. (f) Time‐temperature sweep rheological profiles of SCGs. (g) Crystal‐front propagation velocities of non‐photopolymerized precursor solutions and the corresponding photopolymerized SCGs at different acid content. The acid content was defined as *m_AA_/(m_AA_+m_AAm_)×*100%. (h) Synchronous and asynchronous generalized 2D correlation infrared spectra of SCGs in the 1625–1500 cm^−1^ region under temperature perturbations from 30°C to 150°C. (i) Normalized initial‐to‐final volume ratio of SCGs during crystallization under different acidities (*n* = 10). R_0_ and R denote the edge lengths of the cubic specimens before and after crystallization, respectively, and (R_0_/R)^3^ corresponds to V_0_/V.

In situ polarized optical microscopy (POM) confirms this templated growth, showing highly oriented prismatic crystals propagating from the stimulation point (Figure 2c and Figure S2). Porod analysis of isotropic scattering rings in SAXS profiles confirms that crystalline pillars comprise densely packed nanocrystals strongly bound to the polymer framework, with an interfacial layer thickness of ≈0.98 nm (Figure 2d) [41]. X‐ray diffraction (XRD) verifies the crystalline phase as sodium acetate trihydrate (PDF#29‐1160), with an average crystallite size of 37.97 nm (Figure S3). Such nanoscale dimensions not only promote cascade nucleation through high surface energy but also optimize the effective volume for potential functional payloads. Atomic force microscopy (AFM) was used to characterize the surface topography directly. The transition from an amorphous hydrogel to a crystalline pillar array with ≈450 nm slits (Figure 2e) visually confirms the micro‐scale lattice interlocking responsible for macroscopic stimulus‐stiffening.

The sensitivity of this kinetic unlocking mechanism was quantitatively evaluated via pressure‐triggered assays. Remarkably, minute mechanical perturbations, spanning a low‐pressure regime of 170 to 1638 Pa, consistently induced cascade nucleation (Figure S4). This highlights the exceptional sensitivity of the response, validating the biological “positive‐gain” archetype where minimal kinetic input reliably actuates profound microstructural evolution. However, this direct‐contact sensitivity can be mitigated by dispersing localized interfacial pressure. Following isolation in rigid glass containers, the SCGs retained their metastable non‐crystalline state during simulated short‐distance transportation, indicating that appropriate packaging can prevent accidental triggering during practical storage and handling (Figure S5).

Once initiated, the propagation kinetics of the crystalline front are programmable through environmental and architectural modulation. The crystal‐front velocity exhibited a non‐monotonic temperature dependence, peaking at 35°C before decreasing at higher temperatures, possibly due to competition between enhanced ionic mobility and the reduced thermodynamic driving force at lower degrees of supercooling (Figure S6). As shown in Figure 2g, crystal‐front propagation was compared between non‐photopolymerized precursor solutions and the corresponding photopolymerized SCGs. The precursor controls contained the same components and AAm/AA feed ratios but were not exposed to UV irradiation. Therefore, AAm and AA remained predominantly in their monomeric forms, and no covalently crosslinked polymer network was formed. Relative to these precursor controls, the pristine PAM network functioned as a kinetic buffer, substantially suppressing the otherwise rapid crystallization‐front propagation. By copolymerizing graded amounts of acrylic acid (AA) into the PAM framework, we further tuned the interfacial interactions between the polymer scaffold and the supercooled clusters. Together, polymer‐network formation and AA incorporation enabled graded control over the crystal‐front propagation velocity from 0.043 to 1.786 mm s^−1^. Composition screening further showed that the NaAc‐to‐total‐polymerizable‐monomer feed ratio governed both mechanical reliability and thermal reconfigurability. At a ratio of 6:0.5, two of three independently prepared crystallized specimens exhibited premature mechanical instability at approximately 2% strain, indicating poor reproducibility at the lower polymer content. In contrast, increasing the ratio to 6:2 resulted in incomplete lattice disassembly even at 85°C, as evidenced by the residual crystalline opacity. The 6:1 formulation was therefore selected because it combined reproducible mechanical integrity with complete thermal lattice disassembly (Figure S7).

Despite such rapid kinetics, the phase transition remains a fully reversible thermodynamic process. Temperature‐sweep rheological profiling (Figure 2f) reveals systematic fluctuations in the loss factor (*tanδ*), which accurately maps the temperature‐dependent disintegration and reconstruction of the crystalline lattice. This substantiates that the adaptive switching operates as a switchable thermodynamic behavior rather than an irreversible degradation. Complete cation substitution further showed that the Ca^2+^ formulation remained noncrystalline and soft, whereas the Mg^2+^ formulation crystallized spontaneously upon cooling; neither reproduced the triggerable stiffening of the NaAc‐based SCGs (Figure S8). This comparison indicates that SCG formation requires matching polymer–ion interactions with the salt‐specific solubility and metastable crystallization behavior, rather than simply increasing cation–polymer coordination strength. Together with the reported PAAm/CaCl_2_·6H_2_O phase‐change gel, these results suggest that polymer–salt‐hydrate coupling can be extended to other material systems, although the attainable switching behavior remains governed by salt‐specific phase properties [42].

To quantify the rigid‐to‐soft transition, whole‐specimen thermal lattice disassembly and water‐assisted radial lattice disassembly were evaluated using the loss of crystalline opacity as a structural marker. Increasing the temperature from 55 to 85°C reduced t_50_ from 17.68 ± 2.50 to 1.66 ± 0.36 min and t_90_ from 38.30 ± 1.97 to 3.73 ± 0.80 min. For 25‐mm‐diameter discs, water exposure produced outside‐in radial disassembly, with t_50_ and t_90_ of 57.6 ± 2.7 and 108.6 ± 0.3 min, respectively (*n* = 3; Note S1, Figures S9 and S10).

To investigate the underlying supramolecular interactions, temperature‐dependent FTIR spectroscopy was employed (Figure S11). The significant attenuation of the O─H stretching band (3443–3285 cm^−1^) and the distinct blue‐shifting of the “free” ─COO^−^ (from 1583 cm^−1^) and “bound” ─COO^−^ (from 1540 cm^−1^) peaks confirm the establishment of a multi‐site hydrogen‐bonded network upon crystallization [43]. Perturbation‐correlation moving window (PCMW2D) analysis further reveals that these hydrogen bonds are highly active within a specific thermal window of 30°C to 60°C, underpinning the structural reversibility. The fundamental logic of this templated growth was further deciphered via generalized two‐dimensional correlation spectroscopy (2DCOS) (Figure 2h). Noda's rules reveal a distinct sequential evolution during lattice disintegration: the generation of “free” ─COO^−^ groups strictly precedes the dissociation of “bound” ─COO^−^ groups (Note S2). Physically, this implies that while the internal crystalline order of the salt clusters begins to fluctuate, the heterophase “anchoring” points between the salt and the PAM framework possess superior stability [44]. This “last‐to‐break” behavior suggests that persistent interfacial contacts may help retain a pre‐nucleation ionic environment during the reverse transition. Atomistic molecular dynamics (MD) simulations further show that PAM preserves the primary Na^+^–water/acetate coordination motifs while modestly restricting water and acetate mobility (Figures S12–S16 and Note S3). These results support a kinetic‐confinement mechanism in which PAM limits the cooperative ion–solvent rearrangement required for spontaneous nucleation, rather than acting as a dominant Na^+^ ligand.

This “template‐prior” molecular interlocking successfully decouples the conventional trade‐off between modulus enhancement and macroscopic shape distortion. Unlike conventional phase‐change materials that exhibit drastic expansion during phase transitions, SCGs undergo a macroscopic volumetric change of < 3% (Figure 2i). This extraordinary dimensional stability is attributed to the templated confinement of the polymer network, which internally accommodates the micro‐scale lattice reorganization and buffers the bulk expansion. To further elucidate this stabilization mechanism, we adjusted the acidity of the polymer framework to inhibit the ordered growth of crystalline bundles. The resulting incomplete crystallization led to a significant macroscopic expansion of approximately 7%, contrasting sharply with the stabilized SCGs. This underscores that the templated “climbing” and integration of crystals onto the polymer network are critical for suppressing uncontrolled growth and maintaining structural integrity. Furthermore, the structural stability post‐crystallization is also demonstrated by dehydration resistance. The dense crystalline lattice effectively immobilizes internal solvated molecules, resulting in a negligible mass loss of only 0.2% after 7 days (Figure S17). This confirms that the crystallized state firmly locks the internal solvent, guaranteeing long‐term environmental and structural stability.

### 2.3 Mechanical Adaptation Behavior and Simulation

The stimulus‐triggered lattice formation endows SCGs with robust positive‐gain mechanical adaptation, enabling rapid switching across divergent mechanical platforms. This macroscopic stiffening is established through the site‐specific growth of oriented lattices. Similar to the biomineralization of dental enamel, the SCGs exhibit a templated crystallization that yields superior lattice alignment and crystal regularity [45]. At the point of maximum crystallization tendency (as delineated in the global phase stability map), the SCGs achieve a peak compressive modulus of 116.8 MPa, a 2596‐fold (≈260 000%) amplification relative to the metastable gel state (0.045 MPa) (Figure 3a,b). Notably, this mechanical trajectory is programmable. By modulating the framework acidity via gradient acrylic acid copolymerization, the stiffened state can be tuned within a range of 33.9 to 116.8 MPa (Figure 3c). Complementarily, the SCGs exhibit a “soft‐on‐demand” logic, where the rigid lattice can be selectively hydrolyzed or thermally disintegrated back into the high‐entropy gel state, maintaining a minimal modulus baseline for seamless tissue integration. Water‐assisted disassembly is accompanied by mass exchange and permanent deformation, defining a single‐use device pathway, whereas closed‐system thermal disassembly preserves composition and enables triggered recrystallization. The compressive modulus remained comparable after one cycle but declined to 82.3% and 50.7% of its initial value after 3 and 10 cycles, respectively, demonstrating repeatable reactivation with finite mechanical durability (Figure S18).

**FIGURE 3:**
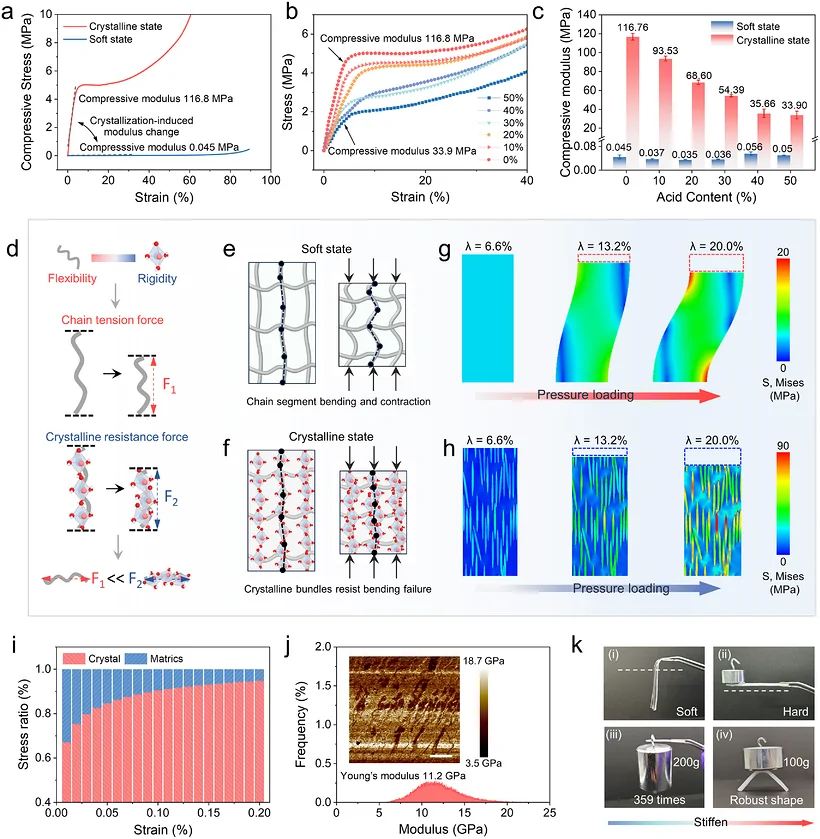
Mechanical properties and adaptation of SCGs. (a) Compressive stress–strain curves of SCGs before and after lattice formation in the absence of acidity regulation. (b) Compressive moduli before and after crystallization under varying acidity‐regulated conditions. (c) Compressive stress–strain curves post‐lattice formation under varying acidity‐regulated conditions. (d) Enhanced buckling resistance of crystalline bundles enabled by oriented crystal alignment. *F_1_* and *F_2_* represent the buckling force of metastable flexible segments and the bending force of crystalline bundles, respectively. (e, f) Schematic illustration of segmental microregions under stress, contrasting (e) polymer chain sliding in the gel state with (f) brittle fracture in the lattice state. (g, h) FEA stress nephograms (distribution maps) (g) without and (h) with oriented lattices under different compression strains. Here, λ denotes the applied compressive strain. (i) Calculated compressive load‐bearing ratios of the matrix and crystalline columns derived from the stress nephograms. (j) AFM modulus mapping and the corresponding modulus distributions of SCGs. Scale bar, 1 µm. (k) Photographs demonstrating the mechanical robustness of SCGs in the lattice state during operation.

The fundamental shift in mechanical impedance originates from the reconfiguration of the internal phase structure. Within the classical polymer physics framework, the metastable PAM scaffold, lubricated by solvated clusters, operates via entropic elasticity where external loads are primarily countered by chain tension and segmental sliding (Figure 3d,e) [46]. Conversely, post‐transformation, the framework is epitaxially interlocked by oriented crystalline bundles. This creates a “structural locking” effect where the crystalline resistance force dominates. The rigid crystalline bundles intrinsically arrest conformational rearrangements such as chain folding or buckling, instead resisting stress through brittle fracture and high Euler buckling resistance (Figure 3f). To quantitatively elucidate this lattice‐mediated mechanics, finite element analysis (FEA) was employed to model an elastic framework embedded with oriented crystalline bundles. Stress‐dependent simulations demonstrate that the in situ formed lattices function as high‐modulus “hard domains” that efficiently intercept and propagate stress (Figure 3h). In contrast, the absence of these oriented bundles leads to a dramatic decline in stress tolerance (Figure 3g). Numerical analysis of the load distribution reveals that the crystalline bundles progressively bear an increasing proportion of the compressive load as strain increases, validating the “positive‐gain” reinforcement logic (Figure 3i). This was further corroborated by AFM nanomechanical mapping, which recorded an average Young's modulus of 11.2 GPa for the intact lattices, dramatically exceeding the stiffness of the human stratum corneum (≈3.5–1000 MPa), the most rigid layer of the skin (Figure 3j). At the macroscopic scale, this adaptive logic translates into exceptional structural robustness: while the soft state behaves as typical compliant hydrogels, the stiffened state exhibits formidable buckling resistance, capable of supporting loads exceeding 359 times its own weight (Figure 3k).

### 2.4 Biomedical Engineering Application

To validate the translational potential of this positive‐gain mechanical adaptation, we engineered microneedle arrays based on supercooled crystal‐gels (SCG‐MNs). Bio‐integrated penetrative devices inherently face a profound physical paradox: they necessitate formidable initial rigidity to breach the stratum corneum, yet demand post‐insertion structural compliance to conform to tissue biomechanics and facilitate rapid mass transport [47]. Recently reported biomolecular‐glass microneedles address these sequential requirements using a crystallization‐resistant amorphous matrix that provides penetration strength, preserves labile biomolecules, and subsequently dissolves after insertion [48]. In contrast, SCG‐MNs exploit mechanically triggered crystallization and water‐assisted lattice disintegration to execute a programmable “soft‐stiff‐soft” mechanical sequence. Leveraging the metastable gel precursor, SCGs were reliably cast into uniform 10 × 10 needle arrays (500 µm base width, 1600 µm height) (Figure 4a, Figure S19 and Note S4). SEM images showed that macroscopic structural integrity was maintained even at a casting hydration of ≈41.7% (Figure S20). Upon triggered crystallization, the stiffened SCG‐MNs exhibit a robust fracture force of 2.28 N/needle, significantly exceeding the threshold required for successful epidermal penetration (0.05 N/needle). Moreover, a representative SCG‐MN patch retained a fracture force sufficient for epidermal penetration after approximately 480 days of sealed ambient storage, supporting the long‐term mechanical stability of the rigid state (Figure S21). Crucially, upon epidermal insertion, physiological moisture dynamically induces water‐assisted lattice disintegration. This environmental trigger unlocks the rigid structure, transitioning the SCG‐MNs into a softened hydrogel state (fracture force: 0.68 N) that seamlessly matches soft tissue mechanics to prevent in vivo fragmentation (Figure 4b,c). After removal from ex vivo porcine skin, 292 of 300 microneedles retained optically intact profiles across three independent patches (97.3 ± 0.6%), demonstrating high macroscopic structural retention during patch removal (Figures S23 and S24). Although no macroscopic residues were observed, microscopic remnants cannot be excluded by optical imaging and require further high‐resolution evaluation for clinical translation.

**FIGURE 4:**
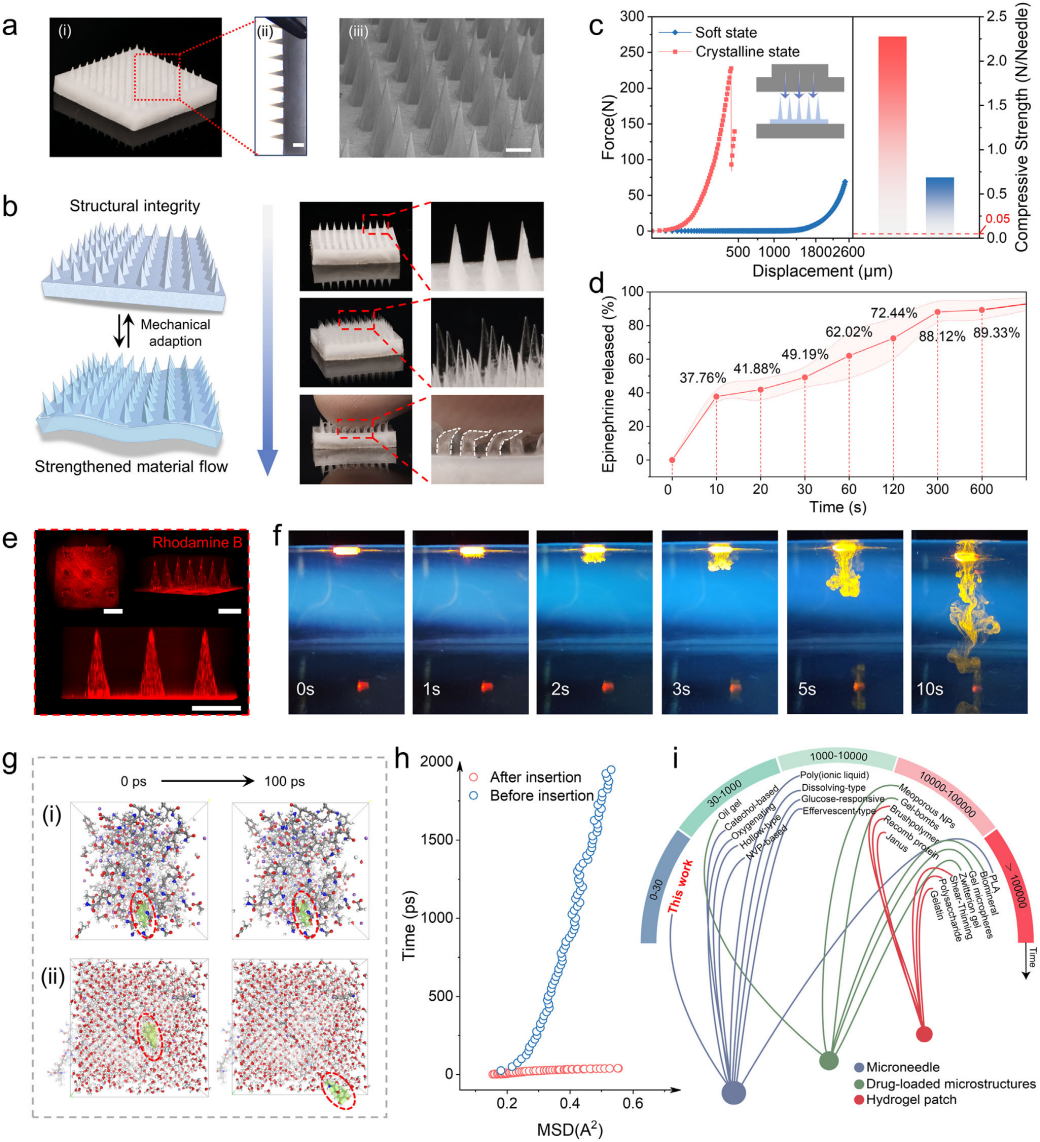
Proof‐of‐concept demonstration of SCGs for adaptive MNs. (a) Structural characterization of fully crystalline SCG‐MNs: (i) digital photograph, (ii) side‐view micrograph, and (iii) SEM image. Scale bars: (ii) 1 mm and (iii) 500 µm. (b) Schematic and photographs of the mechanical adaptation in SCG‐MNs driven by water‐assisted lattice disintegration. (c) Compressive force–displacement curves of SCG‐MNs before and after crystallization, with corresponding fracture forces for individual needles. (d) In vitro cumulative release profiles of epinephrine (10 mg load) from SCG‐MNs in simulated physiological fluid. The light‐colored bands represent the error bands, *n* = 6. (e) Confocal laser scanning microscopy (CLSM) images of SCG‐MNs loaded with RhB‐epinephrine. Scale bar, 1 mm. (f) Visualization of drug release from RhB‐epinephrine‐loaded SCG‐MNs under 365 nm UV excitation. (g) Molecular dynamics (MD) snapshots depicting epinephrine diffusion within SCG‐MNs following lattice disintegration: (i) without and (ii) with liquid‐assisted propulsion. (h) Time‐dependent MSD curves of epinephrine before and after SCG‐MNs insertion into the skin. (i) Comparison of the time required for 50% cumulative drug release (*T_50%_*) between SCG‐MNs and other recently reported advanced drug delivery platforms.

This lattice‐mediated mechanical unlocking concurrently revolutionizes the mass transport profile of the polymer matrix. Conventional gel‐based drug carriers suffer from heavily retarded diffusion due to dense polymeric entanglement [12]. In contrast, the disintegration of crystalline bundles rapidly opens interstitial transport channels, triggering an explosive payload release. In simulated physiological fluids, the delivery efficiency reached ≈37.76% within merely 10 s, sufficient to hit therapeutic windows for emergency interventions (Figure 4d). The cumulative release soared to 88.12% within 5 min, effectively circumventing the release ceiling of traditional hydrogels that typically require days. By adopting the 50% cumulative release time (T_50%_) as a benchmark for release kinetics, the SCG‐MNs are positioned within the rapid‐release range of contemporary delivery platforms (Figure 4i and Table S1). To improve comparability, an endpoint‐aware analysis was further performed against ten microneedle systems designed for rapid delivery while retaining their original endpoint definitions (Figure S25, Table S4, and Note S5).

To elucidate the underlying mechanism driving this seconds‐scale mass transport, we visualized the release kinetics using Rhodamine B (RhB)‐epinephrine under 365 nm UV excitation (Figure 4e). The spatiotemporal mapping reveals a transient lattice disintegration phase (0–2 s), immediately followed by an explosive, large‐scale spatial diffusion of the payload (3–10 s) (Figure 4f). Atomistic molecular dynamics (MD) simulations further decipher this highly accelerated transport behavior. In a solvent‐deprived (intact lattice) state, epinephrine molecules exhibit negligible displacement over a 100‐ps trajectory (Figure 4g‐i). However, upon water‐assisted lattice disintegrations, the sudden influx of solvent molecules induces a liquid‐assisted propulsion effect, driving rapid diffusion toward the containment boundaries (Figure 4g‐ii). Consequently, the Mean Squared Displacement (MSD) analysis reveals that the solvent‐driven lattice disintegration reduces the temporal threshold for equivalent molecular displacement activity by ≈50‐fold (Figure 4h). This confirms that the release in SCG‐MNs is governed by dynamic structural unlocking rather than stochastic polymer‐network diffusion, fundamentally bridging the gap between superior mechanical penetration and ultrafast molecular delivery.

The programmable phase transition of SCGs was further validated by their ability to execute the “soft‐stiff‐soft” mechanical logic within a dynamic physiological environment (Figure 5a). To this end, the transdermal delivery and emergency intervention capabilities of SCG‐MNs were systematically evaluated in vivo. Initial L929 cell assays showed maintained cytocompatibility at SCG extract concentrations of 1–5 wt.% (Figure S26). Because approximately 89.3% of the payload was released within 10 min, this period represents the practical application window of SCG‐MNs. Ion chromatography and high‐performance liquid chromatography measurements showed that the 10‐min NaAc‐derived Na^+^ and residual acrylamide concentrations corresponded to approximately 1.1 and 0.44 wt.% SCGs, respectively, in the 24‐h extraction series. These leachate levels therefore fell within the low‐concentration cytocompatible range observed in the cell assays (Note S6, Figure S27 and S28). Subsequent macroscopic and histological evaluations in rat, rabbit, and miniature pig models confirmed that the stiffened crystalline lattice successfully and uniformly breached the tough epidermal barriers during insertion tests (Figure 5b). Crucially, post‐insertion observations revealed no fragmented needle residues within the tissue. This physically substantiates that the water‐assisted lattice disintegration rapidly softens the matrix in vivo, neutralizing the risk of brittle fracture and ensuring intact, compliant extraction post‐treatment.

**FIGURE 5:**
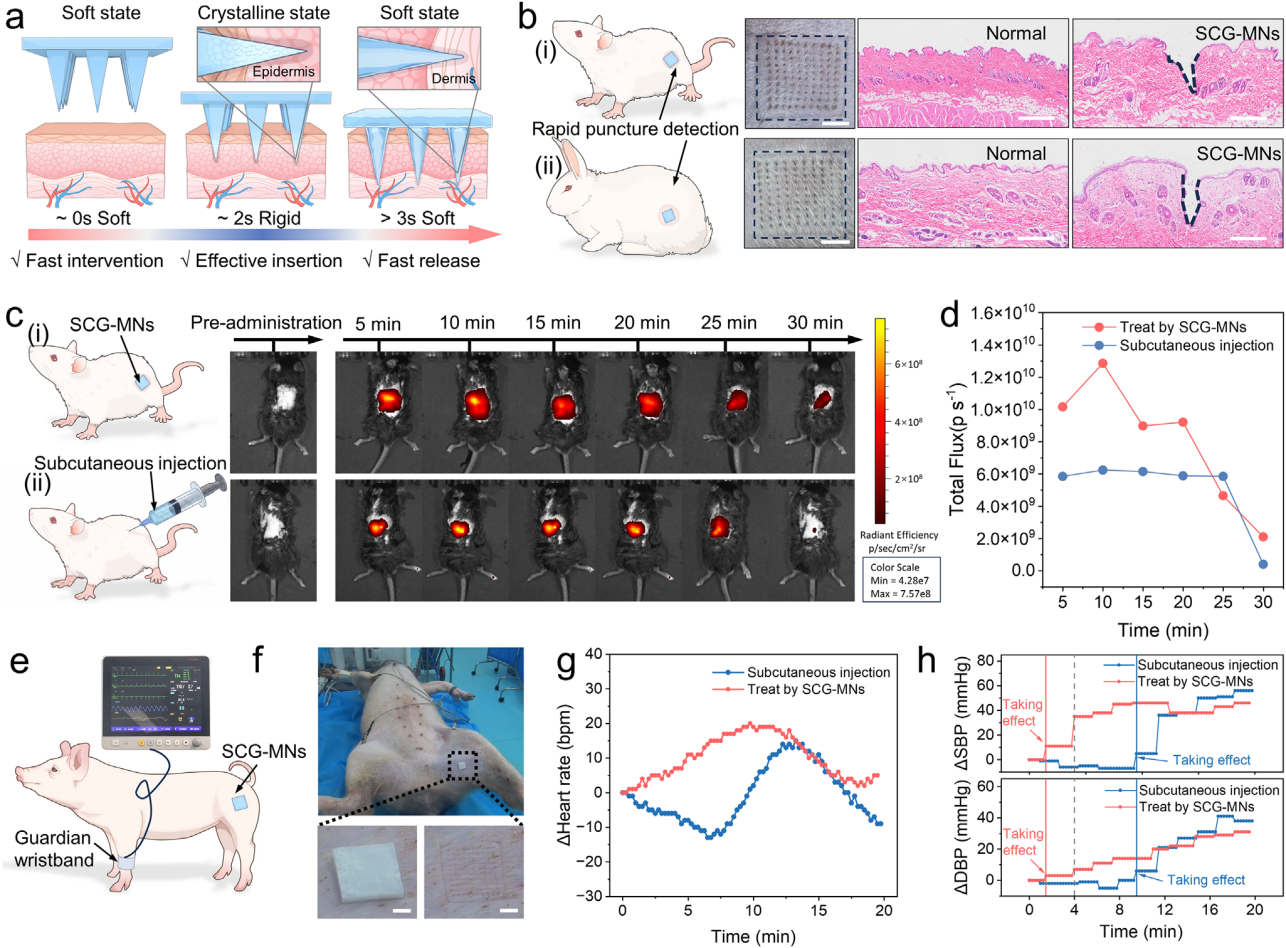
In vivo transdermal delivery and emergency resuscitation using adaptive SCG‐MNs. (a) Schematic illustration of the SCG‐MNs adapting to stage‐specific mechanical requirements during transdermal penetration, enabling effective intervention within seconds. (b) Rapid transdermal evaluation in rat and rabbit models. Digital photographs of the patch applied to (i) rat and (ii) rabbit skin (Scale bar, 4 mm), alongside corresponding hematoxylin and eosin (H&E) stained histological sections before and after SCG‐MNs insertion (Scale bar, 400 µm). (c) In vivo imaging of mice at predetermined time points following (i) application of SCG‐MNs (1 mg RhB‐epinephrine payload) and (ii) subcutaneous injection (0.5 mL solution containing 1 mg RhB‐epinephrine). (d) Time‐dependent quantification of the total fluorescence/bioluminescence intensity. (e) Schematic of the emergency blood pressure resuscitation protocol using MNs in a miniature pig model. (f) Digital photographs capturing the real‐time blood pressure monitoring and subsequent treatment via epinephrine‐loaded SCG‐MNs in the miniature pig. (g, h) Real‐time monitoring of (g) heart rate fluctuations and (h) systolic (SBP) and diastolic blood pressure (DBP) recovery profiles in miniature pigs, comparing the SCG‐MNs and subcutaneous injection modalities (1 mg epinephrine equivalent dose for both).

The translation of this structural unlocking into ultrafast mass transport was first mapped via RhB‐epinephrine fluorescence diffusion in murine models. After acquisition of the pre‐administration baseline image, fluorescence distribution was monitored at 5 min intervals for 30 min following drug administration. Compared to the localized pooling observed in conventional subcutaneous injection controls (Figure 5c), the SCG‐MNs exhibited a vastly expanded initial fluorescence area and prolonged retention. Total luminescence quantification (Figure 5d) confirmed that the SCG‐MNs achieved superior spatial diffusion. This proves that the liquid‐assisted propulsion, triggered by the disintegration of the dense hydrogen‐bonded crystalline bundles, effectively overcomes the tissue diffusion barriers that conventionally delay bolus injections.

To decisively demonstrate the clinical superiority of this non‐equilibrium adaptive material, we assessed its efficacy for rapid blood pressure modulation in a miniature pig model (Figure 5e,f). In severe anaphylaxis or cardiac emergencies, intervention within the “4‐min golden window” is critical for survival [49]. Upon transdermal application of the SCG‐MNs (1 mg epinephrine equivalent) under anesthesia, electrocardiography monitoring recorded an immediate reactive elevation in heart rate (Figure 5g). Remarkably, the SCG‐MNs initiated a significant and sustained elevation in both systolic (SBP) and diastolic blood pressure (DBP) within merely ≈1.5 min (Figure 5h). This ultrafast systemic response comfortably undercuts the critical 4‐min threshold. Conversely, the subcutaneous injection controls suffered from severe localized diffusion delays, requiring ≈9 min to initiate a comparable physiological response. The marginally lower absolute pressure plateau of the SCG‐MNs correlates predictably with residual payload retention within the patch baseline. Collectively, these in vivo dynamics confirm that the kinetically managed phase transition in SCGs paves the way to redefine the temporal limits of soft bio‐integrated electronics, bridging macroscopic structural adaptation with life‐saving molecular intervention.

## 3 Conclusion

In summary, inspired by the tactile‐triggered stiffening of starfish tissues, we have developed dynamic supercooled crystal‐gels that achieve autonomous, positive‐gain mechanical adaptation by harnessing the kinetic modulation of a pre‐stored, non‐equilibrium energy landscape. By kinetically entrapping a supercooled state within a metastable polyacrylamide framework, the system maintains high‐entropy perceptual sensitivity. Upon minute mechanical triggering, this framework acts as a topological scaffold to guide the localized release of latent heat into a templated cascade crystallization. This templated molecular interlocking mechanism successfully decouples the fundamental trade‐off between substantial mechanical reinforcement and dimensional stability, realizing a seconds‐scale, massive modulus amplification while strictly constraining macroscopic volumetric expansion. As a proof of concept, the programmable “soft‐stiff‐soft” mechanical logic of SCGs resolves the mechanical‐transport paradox in bio‐integrated devices, enabling microneedles to achieve tough epidermal penetration followed by ultrafast, liquid‐propelled molecular delivery. This non‐equilibrium kinetic management strategy delineates a generalized paradigm for engineering structural plasticity and functional autonomy, paving the way for next‐generation soft robotics and intelligent physiological interfaces.

## 4 Experimental Section

### 4.1 Reagents and Materials

α‐Ketoglutaric acid (≥ 99%), acrylamide (≥ 99%), and N,N′‐methylenebisacrylamide (MBA, ≥ 99%) were obtained from Sigma–Aldrich (USA) and used as received. Sodium acetate (suitable for molecular biology, ≥ 99%), Calcium acetate (monohydrate, ≥ 99%), and magnesium acetate (tetrahydrate, ≥ 99%) were purchased from Adamas Life (China). RhB–epinephrine was obtained from Solarbio Technology Co., Ltd. (China). Deionized water (resistivity: 18.3 MΩ cm) was used for all experiments.

### 4.2 Fabrication of Supercooled Crystal‐Gels and Microneedle Arrays

The dynamic supercooled crystal‐gels were synthesized via in situ photopolymerization to construct the metastable polymer framework. Initially, pharmaceutical‐grade sodium acetate, acrylamide, and deionized water were mixed at a mass ratio of 6:1:5. The precursor was incorporated with 0.1 wt.% α‐ketoglutaric acid as the photoinitiator and 0.1 wt.% MBA as the cross‐linker. The mixture was heated and continuously stirred at 65°C for 20 min to yield a transparent, metastable polymerizable solution. For drug‐loaded formulations, therapeutic agents were dissolved into the solution strictly below their aqueous solubility limits. The prepolymer was then cast into a polydimethylsiloxane (PDMS) microneedle mold and subjected to a vacuum for 1 min to ensure complete cavity filling and the removal of trapped bubbles. The assembly was subsequently irradiated under UV light (60 W, 365 nm) for 15 s, initiating the polymerization of the polyacrylamide (PAM) framework that kinetically entraps the metastable supercooled state. The final SCG‐MNs were either immediately triggered for lattice solidification (stored in the rigid state in sealed packaging under ambient conditions) or preserved in their soft, metastable state under anoxic conditions (80% RH, room temperature). Feed‐composition screening: SCGs were prepared at NaAc:total polymerizable monomer:water feed mass ratios of 6:0.5:5, 6:1:5, and 6:2:5 using otherwise identical heating and photopolymerization conditions. The compressive behavior of the crystallized 6:0.5:5 specimens was evaluated using three independently prepared samples. To compare thermal lattice disassembly, fully crystallized 6:1:5 and 6:2:5 specimens were sealed to prevent solvent loss and heated at 85°C for 1 h, after which residual crystalline opacity was recorded.

### 4.3 Preparation of Cation‐Substituted Formulations

For complete cation substitution, sodium acetate was replaced with calcium acetate or magnesium acetate at their near‐saturation concentrations at 65°C, while the AAm‐to‐water mass ratio was maintained at 1:5. On an anhydrous‐equivalent basis, the salt: Aam: water mass ratios were approximately 1.6:1:5 and 4.2:1:5 for the calcium‐ and magnesium‐acetate formulations, respectively. All other heating and photopolymerization conditions were identical to those used for the NaAc‐based SCGs. After cooling to room temperature, the samples were mechanically stimulated, and their qualitative deformation and post‐loading shape recovery were recorded.

### 4.4 Structural and Morphological Characterization

Thin sections for lattice observation were prepared by injecting polymerizable solution into a 0.2‐mm‐spaced glass slide sandwich configuration, followed by photopolymerization. All other characterizations employed intact SCG patches. Optical and polarized light microscopy images were acquired using a Phenix XSP‐24 microscope (China) equipped with cross‐polarizers. Surface lattice topography mapping was performed via PeakForce quantitative nanomechanics (Bruker Icon AFM) with antimony‐doped silicon tips (RTESPA‐300‐30). Fully crystallized SCG patch arrays were imaged by SEM (JEOL JSM‐5900LV, Japan). Scattering data collection utilized a Xeuss 2.0 SAXS instrument (France) with a Cu Kα source (λ = 1.54189 Å, 30 W power). Measurements employed a Rayonix MX225‐HE CCD detector at 2480 mm sample‐to‐detector distance. Subsequently, using the software equipped with the beamline, the 2D CCD image was converted into a relationship curve between the scattered intensity (I) and the scattering vector (q = 4πsinθ/λ, where θ is half the scattering angle and λ is the x‐ray wavelength). Organic polyacrylamide‐crystal interface thickness was calculated through Porod's law:

(1) $$E=\sqrt{2\pi \lambda }$$

where *E* denotes interface layer thickness and *λ* represents the slope of Ln (q^3^ *I*(q)) vs. q^2^ in the high q region.

X‐ray diffraction patterns were recorded between 10° and 60° using a Philips X'Pert diffractometer (Netherlands) with Cu Kα radiation (*λ* = 0.1540 nm). Crystallite dimensions were determined via Scherrer's equation applied to the ten highest peaks:

(2) $$D=\frac{K\cdot \lambda }{\beta \cdot \cos\theta }$$

where *D* indicates crystallite size, *K* is the shape factor (0.89), *β* is the full‐width‐at‐half‐maximum, and *θ* is the diffraction angle.

### 4.5 Dynamic and Kinetic Characterization

Monitoring of supercooled crystal‐gels’ dynamic lattice transformation employed a cylindrical glass container (Ø 7 mm × 35 mm height) filled with a metastable polymerizable solution. Variables used to tune the PAM framework included acrylic acid integration level, polymerization status, and ambient conditions. Trigger force application utilized a spring‐loaded iron needle assembly, with load magnitude recorded via force measurement mode on a Sartorius BSA224S analytical balance (Germany). Crystal front propagation rates were determined through time‐lapse imaging using a smartphone camera.

### 4.6 Temperature‐Dependent Thermal Lattice‐Disassembly Kinetics

Fully crystallized SCG specimens were sealed in identical transparent containers to prevent solvent loss or compositional changes and heated in a water bath pre‐equilibrated at 55°C, 65°C, 75°C, or 85°C. Three specimens were monitored simultaneously at each temperature, and images were collected at predetermined time points until the crystalline opacity disappeared. For each image, the corrected optical signal was calculated as follows:

(3) $$R\;\left(t\right)=\frac{I_{s}\left(t\right)}{I_{b}\left(t\right)}$$

where *I*_s_ and *I*_b_ denote the sample and same‐frame background ROI intensities, respectively. The normalized thermal disassembly fraction was calculated as:

(4) $$D_{T}\;\left(t\right)=\frac{\left[R\left(0\right)-R\left(t\right)\right]}{\left[R\left(0\right)-R\left(t_{\mathrm{end}}\right)\right]}$$

Each specimen was descriptively fitted using:

(5) $$D_{T}\;\left(t\right)=1-\;\exp\left[-{\left(\frac{t}{\tau }\right)}^{\beta }\right]$$

The characteristic transition time was obtained from:

(6) $$t_{p}=\tau {\left[-\ln\left(1-p\right)\right]}^{\frac{1}{\beta }}$$

where p = 0.5 and 0.9 for *t*_50_ and *t*_90_, respectively. Data are reported as mean ± SD (*n* = 3 specimens per temperature).

### 4.7 Water‐Assisted Radial Lattice‐Disassembly Kinetics

Fully crystallized circular SCG discs (25 mm in diameter and 2 mm in thickness) were exposed to PBS at 25°C, with the liquid level aligned with the specimen height. Images were collected at 0, 1, 2, 3, 5, 10, 15, 20, 30, 45, 60, 75, 90, 105, 112, and 120 min. The residual crystalline area, A(t), was quantified using Fiji according to manual‐segmentation method and normalized as:

(7) $$C_{A}\;\left(t\right)=\frac{A\left(t\right)}{A\left(0\right)}$$

The water‐assisted disassembly fraction was calculated as:

(8) $$D_{W}\;\left(t\right)=1-C_{A}\left(t\right)$$

Assuming an approximately circular and concentric residual crystalline region, the equivalent radial retreat was calculated as:

(9) $$x\;\left(t\right)=R\left[1-\sqrt{\frac{A\left(t\right)}{A\left(0\right)}}\right]$$

where R = 12.5 mm. The *t*_50_ and *t*_90_ values were obtained by linear interpolation for each specimen, and the apparent radial retreat rate was obtained by linear fitting over 3–105 min. Data are reported as mean ± SD (*n* = 3 independent specimens).

### 4.8 Mechanical Characterization

Mechanical testing utilized a universal testing machine (Instron 5560, USA, 1000 N load cell). Force‐displacement curves for both freshly crystallized patches and fluid‐immersed specimens (3 s immersion) were acquired at an initial tip‐to‐sensor distance of 2.00 mm. For compressive modulus determination, patches had their backings detached and were sectioned into 12.00‐mm‐diameter discs.

### 4.9 Dimensional Variation Test During Crystallization

Identically sized cubic SCG specimens were prepared using the same mold. The edge length was measured at room temperature before mechanical triggering (R_0_) and after complete crystallization (R) using calibrated image analysis. Assuming approximately isotropic dimensional variation, the initial and final volumes were calculated as V_0_ = R_0_^3^ and V = R^3^, respectively. Accordingly, (R_0_/R)^3^ = V_0_/V represents the normalized initial‐to‐final volume ratio, and the relative volume change was calculated as ΔV/V_0_ = [(R/R_0_)^3^ − 1] × 100% (*n* = 10).

### 4.10 Molecular Dynamics (MD) Simulation

All‐atom molecular dynamics (MD) simulations were performed using the Materials Studio software package to investigate the liquid‐solid phase change process of sodium acetate trihydrate. The crystal structure and atomic information for sodium acetate trihydrate were modeled according to established literature methods. To simplify the system, polyacrylamide molecules with a polymerization degree of 10 were constructed using the built‐in modeling tools in Materials Studio. The COMPASS III force field was applied to all models, followed by energy minimization. Subsequently, the system was equilibrated using NPT ensemble simulations, followed by further relaxation in the NVT ensemble. Production simulations were then conducted to calculate the mean squared displacement (MSD). The same simulation protocol except that the model was loaded with several small molecules of epinephrine and water was employed to simulate the rapid release of drugs. Atomic motion followed classical Newtonian mechanics, integrated using the velocity‐Verlet algorithm with a time step of 1.0 fs. The MSD characterizes the mobility of the selected species: a particle, and can be used to determine the diffusion, transport, and binding states of atoms or molecules:

(10) $$MSD=\left[\frac{\sum_{i=1}^{i=N}{\left|r_{i}\left(t\right)-r_{i}\left(0\right)\right|}^{2}}{N}\right]$$

where *N* is the number of Na^+^, *r_i_* (*t*) is the position of the Na^+^ at time 𝑡 and *r_i_* (0) is the position of the Na^+^ at time 0. After that, choose the data 𝑡 at longer time scales to calculate the diffusion coefficient (*D_MSD_*). The 𝐷_𝑀𝑆𝐷_ is calculated as:

(11) $$D_{\mathrm{MSD}}=\frac{1}{6}\lim_{t\to \infty }\frac{d_{MSD}}{d_{t}}$$

To further resolve the molecular role of PAM, an additional pair of comparative all‐atom MD models was constructed for the concentrated pre‐nucleation NaAc–H_2_O phase with and without PAM. These calculations complement, rather than replace, the crystal‐based phase‐transition and drug‐release simulations described above. The PAM‐free model contained 208 Na^+^ ions, 208 acetate ions, and 789 water molecules in a cubic periodic cell (a = b = c = 33.5895 Å). The PAM‐containing model comprised the same numbers of ions and water molecules together with four linear PAM chains, each containing ten acrylamide repeat units (40 PAM carbonyl oxygen atoms in total), in a cubic periodic cell (a = b = c = 34.7115 Å). Anhydrous sodium acetate was used to construct the ionic aqueous models. To focus on local polymer–ion–water interactions at tractable computational cost, the crosslinked network was represented by finite PAM chains; the low‐content photoinitiator and MBA crosslinker were not included explicitly.

All components were described using the COMPASS III force field with force‐field‐assigned charges. Long‐range electrostatic interactions were treated using Ewald summation, and van der Waals interactions were evaluated using the atom‐based method. After geometry optimization with the Smart algorithm, each model was relaxed at 338 K for 200 ps in the NVT ensemble and equilibrated at 338 K and 1 atm for 500 ps in the NPT ensemble. The systems were then cooled stepwise by 200 ps NPT simulations at 318 and 298 K, followed by a further 300 ps NPT equilibration at 298 K and 1 atm. A 1 ns production trajectory was subsequently collected in the NVT ensemble at 298 K. Atomic motion was integrated with the velocity‐Verlet algorithm using a 1 fs time step; the Nosé and Berendsen methods were used for temperature and pressure control, respectively. Coordinates were saved every 5 ps, yielding 201 frames from 0 to 1000 ps for analysis.

Radial distribution functions (RDFs) were calculated for Na^+^–water oxygen and Na^+^–acetate oxygen pairs in both models and for Na^+^–PAM carbonyl oxygen pairs in the PAM‐containing model. First‐shell coordination numbers were obtained by integrating each RDF to its corresponding first minimum. Mean‐square displacements (MSDs) of Na^+^, acetate, and water were calculated from the 1 ns production trajectories. Apparent diffusion coefficients were obtained from linear fits over the common 100–500 ps interval using the three‐dimensional Einstein relation, MSD(t) = 6Dt + b, and fitting‐window sensitivity was evaluated separately. These additional simulations characterize PAM‐induced changes in pre‐nucleation coordination and molecular mobility; they do not directly reproduce the rare nucleation event or the complete gel‐to‐crystal transition.

### 4.11 Temperature‐Dependent FTIR Spectroscopy

Temperature‐dependent FTIR spectra were acquired on a Nicolet iS50 spectrometer (USA) equipped with a deuterated triglycine sulfate (DTGS) detector. Samples mounted on ZnSe windows underwent temperature ramping from 30°C to 150°C at 5°C/min using a programmable custom‐built heating stage, with spectral collection across 3800–1000 cm^−1^. Baseline correction preceded data analysis. Two‐dimensional correlation spectroscopy (2D‐COS) processing employed 2DCS software implementing Noda's rules. Band shift chronology was interpreted as follows: identical signs of synchronous Φ(*ν*_1_,*ν*_2_) and asynchronous Ψ(*ν*_1_,*ν*_2_) cross‐peaks indicate prior movement of band *ν*_1_ relative to *ν*_2_. Non‐zero synchronous correlation with null asynchronous intensity signifies simultaneous band shifts at *ν_1_* and *ν*_2_.

### 4.12 Finite Element Modeling

Two‐dimensional representative volume element (RVE) models were developed in ABAQUS/Explicit for both pure matrix and crystal‐reinforced composites. The 5 mm × 2 mm RVEs employed 0.01 mm mesh elements (A 4‐node bilinear plane stress quadrilateral, reduced integration with hourglass control). Material models assigned the matrix a hyperelastic constitutive law and crystals an elastoplastic formulation. Periodic boundary conditions enforced in‐plane deformation continuity, with 1 mm displacement loading applied at the top surface and y‐direction constraints at the bottom. The crystal‐reinforced model incorporated 98 randomly oriented crystals (0°–10° relative to the vertical axis), ensuring statistical representativeness at this RVE scale.

### 4.13 Drug Release Rate

To evaluate the in vitro release rate of epinephrine from microneedles, patches containing rhodamine‐labeled epinephrine were incubated in PBS (pH 7.4) at 37°C with gentle agitation. At predetermined time points, the released epinephrine was quantified using a fluorescence spectrophotometer.

### 4.14 Quantification of Leachable Sodium Ions and Residual Acrylamide

SCG specimens were extracted in deionized water at an SCG‐to‐water mass ratio of 1:20 for 10 min or 24 h. The aqueous extracts were collected and diluted with deionized water as required for instrumental analysis. Sodium ions originating from NaAc were quantified by ion chromatography (IC), whereas residual acrylamide (AAm) was quantified by high‐performance liquid chromatography (HPLC). Analyte concentrations were determined using external calibration curves prepared from the corresponding standards, corrected for the respective dilution factors, and reported both as concentrations in the extracts and as mass‐normalized release per gram of SCG.

### 4.15 Skin Tissue Penetration Experiment

All animal experiments were conducted in accordance with the protocol approved by the Medical Ethics Committee of West China Hospital of Stomatology, Sichuan University (Approval Number: WCHSIRB‐AT‐2025‐558).

To assess the transdermal performance of the microneedles, Sprague‐Dawley (SD) rats and New Zealand rabbits were used as subjects. The animals were first anesthetized, and the hair on their backs was removed and disinfected. A 10 × 10 array of microneedles (size: 14 mm × 14 mm) was quickly pressed into the depilated skin and held for 1 min. After penetration, the skin was immediately excised and fixed in 4% paraformaldehyde. After 24 h, the fixed skin tissue was paraffin‐embedded, sectioned, and subjected to hematoxylin‐eosin (HE) staining. Finally, the stained sections were imaged under a microscope to determine the penetration capability of the microneedles.

### 4.16 In Vivo Drug Diffusion Experiment

Six‐week‐old female C57BL/6 mice were anesthetized with isoflurane, followed by drug administration via local injection into the dorsal skin or microneedle application. Rhodamine‐labeled epinephrine was used to evaluate drug retention and diffusion rates, with the total dosage kept consistent between the injection and microneedle groups. A baseline image was acquired before administration. SCG‐MNs were then applied, or the control solution was administered by subcutaneous injection, immediately afterward. Subcutaneous fluorescence images were acquired and quantified at 5, 10, 15, 20, 25, and 30 min using a PerkinElmer in vivo imaging system (IVIS).

### 4.17 In Vivo Application Efficacy of Epinephrine

Parma pigs weighing ∼ 30 kg were selected for in vivo experiments. To ensure a sterile environment and reliable results, the surgical site was shaved and disinfected. A catheter was then connected to a 16‐channel physiological recorder to continuously monitor key clinical parameters, including heart rate and blood pressure. After blood pressure stabilized, epinephrine‐loaded microneedle patches were applied for drug delivery. Control miniature pigs received direct epinephrine injections, and changes in blood pressure and heart rate were monitored. The effectiveness of transdermal epinephrine delivery by the device was evaluated by comparing the changes in blood pressure and heart rate between the two groups.

### 4.18 In Vitro Biocompatibility Test

The cytotoxicity of microneedle patches with different mass fractions was assessed using the CCK‐8 assay. Briefly, 10^4^ L929 cells were seeded into 96‐well plates and cultured at 37°C for 24 h. The cells were then exposed to microneedle patches of varying mass fractions for 1, 3, or 5 days. After treatment, the cells were washed with PBS, and a complete medium containing 10% CCK‐8 was added, followed by incubation at 37°C for 2 h. Absorbance was measured at 450 nm using a multifunctional microplate reader. Additionally, L929 cells were seeded into 6‐well plates (1.5 × 10^5^ cells per well) and co‐cultured with microneedle patches of different mass fractions. After 24 h of incubation, the cells were washed twice with PBS, fixed with 4% paraformaldehyde at room temperature for 10 min, and permeabilized with 0.1% Triton X‐100 for 2 min, followed by PBS rinses. To observe cell morphology and adhesion, samples were stained with tetramethylrhodamine isothiocyanate (TRITC)‐labeled phalloidin (Solarbio, China) according to the manufacturer's instructions, followed by DAPI staining in the dark for 5 min.

## Author Contributions

**Yuesong Lv**: conceptualization, data curation, investigation, Writing – original draft, methodology, validation, visualization, Writing – review and editing, software, formal analysis. **Hao Feng**: conceptualization, investigation, validation, data curation. **Zhangqin Yang**: software, methodology. **Jianping Yin**: validation, visualization. **Hanfei Li**: visualization. **Bo Zhou**: supervision. **Yuyang Meng**: resources. **Yuntong Su**: resources. **Xinxing Zhang**: writing – review and editing, methodology, conceptualization, funding acquisition, investigation. **Xibo Pei**: conceptualization, funding acquisition, resources, supervision, methodology.

## Funding

This work was supported by the National Natural Science Foundation of China (52173112 and 52373116, received by X.Z., 82271016 and 82471021, received by X.P.), the Natural Science Foundation of Sichuan Province (2023NSFSC2000), and the Research and Development Program (West China Hospital of Stomatology, Sichuan University) (Nos. RD‐03202310).

## Conflicts of Interest

The authors declare no conflicts of interest.

## Supporting information




**Supporting File**: advs78566‐sup‐0001‐SuppMat.docx.

## Data Availability

The data that supports the findings of this study are available in the supplementary material of this article.

## References

[1] A. Chen , Z. Wang , Z. Guan , et al., “Echinoderm Stereom Gradient Structures Enable Mechanoelectrical Perception,” Nature 651, no. 8105 (2026): 371–376, 10.1038/s41586-026-10164-9. PMC12979197 41741637

[2] A. Deridoux , S. Heydari , S. N. Gorb , E. Kanso , P. Flammang , and S. Gabriele , “Tube Feet Dynamics Drive Adaptation in Sea Star Locomotion,” Proceedings of the National Academy of Sciences 123, no. 3 (2026): 2509681123, 10.1073/pnas.2509681123. PMC12818564 41528874

[3] B. Yi , H. Li , S. Chen , et al., “Superspreading and Ultra‐Infiltrative Coacervate Mediates Strong Underwater Adhesion on Hydrated and Lipidic Substrates,” Nature Chemistry 18, no. 4 (2026): 790–799, 10.1038/s41557-026-02087-9. 41912784

[4] X. Li , B. Zhou , X. Qiu , et al., “Wood Microchannel‐Confined Assembly of Cellulosic Liquid Crystals for Modulable Chromatic Materials,” Advanced Materials 38, no. 9 (2026): 18709, 10.1002/adma.202518709. 41362272

[5] P. Zhou , G. Jiang , Y. Wang , Y. Tian , and X. Zhang , “Self‐Adaptive and Large‐Area Sprayable Thermal Management Coatings for Energy Saving,” Nature Communications 16, no. 1 (2025): 3791, 10.1038/s41467-025-59259-3. PMC12015516 40263304

[6] C. Ni , D. Chen , Y. Yin , et al., “Shape Memory Polymer With Programmable Recovery Onset,” Nature 622, no. 7984 (2023): 748–753, 10.1038/s41586-023-06520-8. 37704734

[7] S. Wu , S.‐Y. Lee , and Y. Zhu , “Thermally Actuated Soft Robotics,” Advanced Materials 37, no. 41 (2025): 04683, 10.1002/adma.202504683. PMC12531750 40708385

[8] J. Huang , H. Zhang , J. Chen , et al., “Rewriting Polymer Fate via Chemomechanical Coupling,” Advanced Materials 38, no. 24 (2026): 18567, 10.1002/adma.202518567. PMC13113212 41631356

[9] S. Liu , D. S. Shah , and R. Kramer‐Bottiglio , “Highly Stretchable Multilayer Electronic Circuits Using Biphasic Gallium‐Indium,” Nature Materials 20, no. 6 (2021): 851–858, 10.1038/s41563-021-00921-8. 33603186

[10] X. Yang , H. Qie , S. Ma , et al., “Beyond Swelling and Shrinking: Achieving a Quasi‐Isovolumetric Phase Transition in Water‐Driven Thermo‐Responsive Hydrogels via Enthalpy–Entropy Compensation,” Angewandte Chemie International Edition 65 (2026): 9390820. 10.1002/anie.9390820 42095556

[11] S. J. K. O'Neill , Z. Huang , X. Chen , et al., “Highly Stretchable Dynamic Hydrogels for Soft Multilayer Electronics,” Science Advances 10, no. 29 (2024): adn5142, 10.1126/sciadv.adn5142. PMC466958 39018406

[12] F. Zhao , M. Liu , H. Guo , et al., “Stimuli‐Responsive Hydrogels Based on Protein/Peptide and Their Sensing Applications,” Progress in Materials Science 148 (2025): 101355, 10.1016/j.pmatsci.2024.101355.

[13] M. Paatelainen , H. Meteling , A. Berdin , and A. Priimagi , “Live‐Shaping of Hydrogel Thin Films With Light,” Nature Communications 17, no. 1 (2026): 3613, 10.1038/s41467-026-71438-4. PMC13100152 42014393

[14] L. Cera , G. M. Gonzalez , Q. Liu , et al., “A Bioinspired and Hierarchically Structured Shape‐Memory Material,” Nature Materials 20, no. 2 (2021): 242–249, 10.1038/s41563-020-0789-2. 32868876

[15] T. Chen , J. Xu , C. Wang , et al., “Shape‐Memory Polyurethanes for Polar Wearables With Ultrasensitive Multi‐Monitoring,” Nature Communications 16, no. 1 (2025): 11329, 10.1038/s41467-025-66422-3. PMC12722725 41381552

[16] J. Han , H. An , M.‐H. Oh , et al., “Dual‐Mode Magnetic Elastomer for On‐Demand Motion and Degradation,” Advanced Functional Materials 36 (2026): 75790.

[17] H. Xu , S. Wu , Y. Liu , et al., “3D Nanofabricated Soft Microrobots With Super‐Compliant Picoforce Springs as Onboard Sensors and Actuators,” Nature Nanotechnology 19, no. 4 (2024): 494–503, 10.1038/s41565-023-01567-0. PMC11026159 38172430

[18] A. Dana , C. Benson , M. Sivaperuman Kalairaj , et al., “Collective Action and Entanglement of Magnetically Active Liquid Crystal Elastomer Ribbons,” Matter 8 (2025): 102270.

[19] X. Qin , Y. Tian , H. Zhao , et al., “Toward Reprocessable High‐Performance Elastomer: Self‐Assembly, Dynamic Covalent Chemistry, and Tailorable Properties,” Advanced Materials 38, no. 29 (2026): 15432, 10.1002/adma.202515432. 41387234

[20] I. Hwang , S. Mun , J.‐H. Youn , et al., “Height‐Renderable Morphable Tactile Display Enabled by Programmable Modulation of Local Stiffness in Photothermally Active Polymer,” Nature Communications 15, no. 1 (2024): 2554, 10.1038/s41467-024-46709-7. PMC10959967 38519461

[21] C. Xu , C. F. J. Faul , M. Taghavi , and J. Rossiter , “Electric Field‐Driven Dielectrophoretic Elastomer Actuators,” Advanced Functional Materials 33, no. 13 (2023): 2208943, 10.1002/adfm.202208943.

[22] K. Guo , X. Yang , C. Zhou , and C. Li , “Self‐Regulated Reversal Deformation and Locomotion of Structurally Homogenous Hydrogels Subjected to Constant Light Illumination,” Nature Communications 15, no. 1 (2024): 1694, 10.1038/s41467-024-46100-6. PMC10894256 38402204

[23] C. Liang , V. Dudko , O. Khoruzhenko , et al., “Stiff and Self‐Healing Hydrogels by Polymer Entanglements in Co‐Planar Nanoconfinement,” Nature Materials 24, no. 4 (2025): 599–606, 10.1038/s41563-025-02146-5. PMC11961364 40055539

[24] Z. J. Wang , W. Li , X. Li , T. Nakajima , M. Rubinstein , and J. P. Gong , “Rapid Self‐Strengthening in Double‐Network Hydrogels Triggered by Bond Scission,” Nature Materials 24, no. 4 (2025): 607–614, 10.1038/s41563-025-02137-6. PMC12010354 40011594

[25] J.‐Y. Shen , C.‐Y. Shi , T. He , W.‐Y. Qin , H. Tian , and D.‐H. Qu , “Disulfide‐Mediated Confinement Assembly Enabling Thermal‐Hyperhardening Hydrogels via Phase Evolution,” Advanced Materials 38, no. 18 (2026): 23672, 10.1002/adma.202523672. 41778448

[26] Y. Zhang , W. Zhao , S. Ma , et al., “Modulus Adaptive Lubricating Prototype Inspired by Instant Muscle Hardening Mechanism of Catfish Skin,” Nature Communications 13, no. 1 (2022): 377, 10.1038/s41467-022-28038-9. PMC8770490 35046391

[27] Z. Wang , J. Wang , J. Ayarza , et al., “Bio‐Inspired Mechanically Adaptive Materials Through Vibration‐Induced Crosslinking,” Nature Materials 20, no. 6 (2021): 869–874, 10.1038/s41563-021-00932-5. 33619367

[28] T. Matsuda , R. Kawakami , R. Namba , T. Nakajima , and J. P. Gong , “Mechanoresponsive Self‐Growing Hydrogels Inspired by Muscle Training,” Science 363, no. 6426 (2019): 504–508, 10.1126/science.aau9533. 30705187

[29] J. Chen , Z. Wang , B. Yao , et al., “Ultra‐Highly Stiff and Tough Shape Memory Polyurea With Unprecedented Energy Density by Precise Slight Cross‐Linking,” Advanced Materials 36, no. 27 (2024): 2401178, 10.1002/adma.202401178. 38648568

[30] X. Ding , J. Chen , Z. Liu , Z. Liu , and Y. Zhu , “Fast‐Response and Large‐Deformation Moisture‐Driven Janus Structured Fiber Actuator Composed of Multiscale Hydrophilic‐Hydrophobic Interfaces,” Advanced Functional Materials 36, no. 18 (2026): 18322, 10.1002/adfm.202518322.

[31] T. Zhang , D. Zhang , W. Chen , et al., “Shape and Stiffness Switchable Hydroplastic Wood With Programmability and Reproducibility,” ACS Nano 17, no. 23 (2023): 23524–23534, 10.1021/acsnano.3c06322. 38032080

[32] Y. Li , H. H. Tang , Y. C. Luo , et al., “Reversible Stepless Multiple Shape Memory Polymer Comprising Amorphous Region as Switching Phase,” Advanced Functional Materials 35, no. 35 (2025): 2500230, 10.1002/adfm.202500230.

[33] L. Chen , C. Zhao , J. Huang , J. Zhou , and M. Liu , “Enormous‐Stiffness‐Changing Polymer Networks by Glass Transition Mediated Microphase Separation,” Nature Communications 13, no. 1 (2022): 6821, 10.1038/s41467-022-34677-9. PMC9649666 36357428

[34] Z. Huang , Z. Wu , C. Li , et al., “Self‐Healing Yet Strong Actuator Materials With Muscle‐Like Diastole and Contraction via Multilevel Relaxations,” Advanced Materials 37, no. 5 (2025): 2413194, 10.1002/adma.202413194. 39659125

[35] A. D. Crowell , J. Kang , D. L. Conrad , et al., “Leveraging Bond Dissociation Kinetics to Tune Shear‐Thickening Behavior in Dynamic Covalent Tetra‐PEG Hydrogels,” Science Advances 12, no. 10 (2026): adz9563, 10.1126/sciadv.adz9563. PMC12959392 41779839

[36] C.‐Y. Lyu , W. Xia , T. Ma , et al., “A Recyclable Polythioester With α ‐Gem‐Dimethyl Substitution: Instantaneous Crystallization Triggered by Large and Rapid Stretching,” Advanced Materials 38, no. 14 (2026): 21662, 10.1002/adma.202521662. 41641914

[37] J. Mo , S. F. Prévost , L. M. Blowes , et al., “Interfibrillar Stiffening of Echinoderm Mutable Collagenous Tissue Demonstrated at the Nanoscale,” Proceedings of the National Academy of Sciences 113, no. 42 (2016): E6362, 10.1073/pnas.1609341113. PMC5081653 27708167

[38] T. Yang , H. Chen , Z. Jia , et al., “A Damage‐Tolerant, Dual‐Scale, Single‐Crystalline Microlattice in The Knobby Starfish, Protoreaster nodosus,” Science 375, no. 6581 (2022): 647–652, 10.1126/science.abj9472. 35143308

[39] A. Deridoux , S. Heydari , S. N. Gorb , E. A. Kanso , P. Flammang , and S. Gabriele , “Tube feet dynamics drive adaptation in sea star locomotion,” Proceedings of the National Academy of Sciences of the United States of America 123', no. 3 (2026): e2509681123. 10.1073/pnas.2509681123 PMC12818564 41528874

[40] T. B. H. Schroeder and J. Aizenberg , “Patterned Crystal Growth and Heat Wave Generation in Hydrogels,” Nature Communications 13, no. 1 (2022): 259, 10.1038/s41467-021-27505-z. PMC8752664 35017471

[41] Y. Lv , C. Li , Z. Yang , et al., “Monomer Trapping Synthesis Toward Dynamic Nanoconfinement Self‐healing Eutectogels for Strain Sensing,” Advanced Science 11, no. 42 (2024): 2410446, 10.1002/advs.202410446. PMC11558160 39279471

[42] H. Jing , L. Xu , X. Wang , Y. Liu , and J. Hao , “A Phase‐Change Gel Based Pressure Sensor With Tunable Sensitivity for Artificial Tactile Feedback Systems,” Journal of Materials Chemistry A 9, no. 35 (2021): 19914–19921, 10.1039/D1TA02791C.

[43] C. Li , X. Yang , Y. Wang , J. Liu , and X. Zhang , “Core–Shell Nanostructured Assemblies Enable Ultrarobust, Notch‐Resistant and Self‐Healing Materials,” Advanced Functional Materials 34, no. 52 (2024): 2410659, 10.1002/adfm.202410659.

[44] X. Yang , X. Huang , X. Qiu , Q. Guo , and X. Zhang , “Supramolecular Metallic Foams With Ultrahigh Specific Strength and Sustainable Recyclability,” Nature Communications 15, no. 1 (2024): 4553, 10.1038/s41467-024-49091-6. PMC11137098 38811594

[45] H. Zhao , S. Liu , Y. Wei , et al., “Multiscale Engineered Artificial Tooth Enamel,” Science 375, no. 6580 (2022): 551–556, 10.1126/science.abj3343. 35113708

[46] Y. Wang , R. Shu , and X. Zhang , “Strong, Supertough and Self‐Healing Biomimetic Layered Nanocomposites Enabled by Reversible Interfacial Polymer Chain Sliding,” Angewandte Chemie International Edition 62, no. 23 (2023): 202303446, 10.1002/anie.202303446. 37010017

[47] T. Bauleth‐Ramos , N. El‐Sayed , F. Fontana , M. Lobita , M.‐A. Shahbazi , and H. A. Santos , “Recent Approaches for Enhancing the Performance of Dissolving Microneedles in Drug Delivery Applications,” Materials Today 63 (2023): 239–287, 10.1016/j.mattod.2022.12.007.

[48] W. Fan , P. Zhou , G. Shen , et al., “Noncovalent Cluster Packing Enables Ultrastable Glasses for Preservation and Delivery of Labile Biomolecules,” Angewandte Chemie International Edition 65, no. 16 (2026): 4411241, 10.1002/anie.4411241. 41766217

[49] C. P. Allen , J. D. Bird , and M. S. Sekhon , “The Dynamic Pathophysiology of Post Cardiac Arrest Brain Injury: “Time is Brain”,” Current Opinion in Critical Care 31 (2025): 123–130. 10.1097/MCC.0000000000001246 40047231

