# Supplementary material for: Dynamic Supercooled Crystal–Gels for Ultrafast, Reversible Mechanical Adaptative Materials
Source: Adv Sci (Weinh). 2026 Nov 3:e78566. Online ahead of print. doi: 10.1002/advs.78566 (PMC13728868; doi:10.1002/advs.78566)
Supplement: Supplementary file 1 [file ADVS-9999-e78566-s001.docx]

Supporting information for

**Dynamic Supercooled Crystal–Gels for Ultrafast, Reversible Mechanical Adaptative Materials**

Yuesong Lv, Hao Feng, Zhangqin Yang, Jianping Yin, Hanfei Li, Bo Zhou, Yuyang Meng, Yuntong Su, Xinxing Zhang*, Xibo Pei*

Note S1**:** Thermal and Water-Assisted Lattice-Disassembly Kinetics of SCGs

To quantitatively evaluate the rigid-to-soft transition, we characterized the whole-specimen thermal lattice-disassembly kinetics and the water-assisted radial lattice-disassembly kinetics as two distinct softening pathways. The disappearance of the load-bearing crystalline domains was used as an operational structural marker of softening. Under sealed heating, the characteristic *t*_50_ decreased from 17.68 ± 2.50 min at 55 °C to 3.74 ± 0.83, 2.91 ± 0.31, and 1.66 ± 0.36 min at 65, 75, and 85 °C, respectively, while *t*_90_ decreased from 38.30 ± 1.97 to 7.81 ± 0.87, 4.85 ± 0.19, and 3.73 ± 0.80 min, respectively (Figure S9). Thus, heating from 55 to 85 °C shortened both characteristic transition times by approximately 90%, demonstrating strongly temperature-dependent thermal lattice disassembly. Water exposure produced a distinct outside-in disassembly pathway. For 25-mm-diameter SCG discs, the residual crystalline-area fraction yielded t_50_ and t_90_ values of 57.6 ± 2.7 and 108.6 ± 0.3 min, respectively (n = 3 independent specimens). Following the initial wetting period, the equivalent radial retreat progressed approximately linearly between 3 and 105 min at an apparent rate of 0.0506 ± 0.0020 mm min^−1^ (R^2^ = 0.970 ± 0.002), with complete loss of the crystalline region at approximately 112 min (Figure S10). These values describe the geometry-dependent radial disassembly of the macroscopic discs and quantitatively establish the water-assisted softening pathway.

Note S2**:** Analysis of multiple interactions in dynamic crystallization

To elucidate interaction mechanisms, temperature-dependent FTIR spectroscopy was carried to characterize hydrogen-bond evolution during the lattice disintegration process of SCGs. As shown in Figure S11a,b, the O–H stretching band (3443–3285 cm^-1^) exhibits significant attenuation. The rising peak assigned to “free” –COO^-^ groups at 1583 cm^-1^ displays marked blue-shifting, and the declining “bound” –COO^-^ peak at 1540 cm^-1^ similarly blue-shifts. These observations confirm multi-site hydrogen bonding in the crystallized SCGs. Perturbation−correlation moving window two-dimensional (PCMW2D) correlation spectroscopy was used to investigate the precise dissociate temperature of hydrogen bonds. As illustrated in Figure S11c,d, hydrogen bonds are highly active over 30 to 60 °C, demonstrating the reversibility of bonding and structure. Furthermore, interaction dissociation sequence was probed by two-dimensional correlation spectra to analyze lattice disintegration. Spectral intensity change order at given wavenumbers can be determined according to Noda’s rules based on the sign of the correlated peaks in the synchronous and asynchronous spectra. The generalized 2D correlation FTIR spectra (1500–1625 cm^-1^; Figure.2h, S11) reveal that “free” –COO^-^ generation precedes “bound” –COO^-^ dissociation. This indicates that lattice disintegration proceeds through molecular-scale crystal fragmentation rather than bulk crystal detachment, with –COO^-^ groups maintaining interactions with the polymer framework.

Note S3**:** Molecular dynamic simulation

All-atom molecular dynamics (MD) simulations were used to explore the liquid-solid phase change process of sodium acetate trihydrate. The MD calculation process was carried out in Materials Studio (MS) software package. In the MD simulations, the crystal structure of sodium acetate trihydrate was modeled by creating the crystal structure and atomic information following the methods in literature. The specific crystal structure information of single crystal unit is as follows: monoclinic crystal system is selected, space group is 2C/c, lattice length (a: 12.353 Å, b: 10.466 Å, c: 10.401 Å), lattice Angle β is 111.69°. In order to simplify the calculation, polyacrylamide molecules with polymerization degree of 10 were established using the built-in modeling system of MS. The force field was assigned using COMPASS (Condensed Phase Optimized Molecular Potentials for Atomistic Simulation Studies) III. COMPASS III force field can accurately predict the molecular structure, conformation, vibration and thermodynamic properties of isolated and condensed molecules, which is used to describe the interactions between organic molecules. After the calculations were completed, all models were optimized to optimize the energy and force tolerances of the two systems to 0.001 kcal mol^-1^ and 0.5 kcal mol^-1^ Å^-1^, respectively. Subsequently, a 100ps of MD simulations were performed for the NPT set at temperatures 298K and an enclosing pressure of 1e^-4^ GPa, and a 100 ns of MD simulations were then performed for the NVT set at temperatures 298K to further relax the system. The above two simulation steps are 1fs, Nosé–Hoover method and the Berendsen method were used for temperature and pressure control. Atom and Ewald methods were used for Van der Waals and electrostatic interactions, respectively. Finally, production simulations were performed to calculate the MSD by the system. The same is true for simulating the rapid release of drugs, except that the above model is loaded with several small molecules of epinephrine and water. The atomic motion in the system follows classical Newtonian motion, where a velocity-Verlet algorithm with a time step of 1.0 fs is used to integrate the classical Newtonian equations.

To clarify the molecular role of PAM before nucleation, an additional pair of all-atom models was constructed for concentrated NaAc–H_2_O systems with and without four linear PAM chains (degree of polymerization = 10). Both systems underwent the same equilibration protocol, followed by a 1 ns NVT production run at 298 K. RDF analysis showed that PAM did not substantially alter the native Na^+^ coordination shell: the first maxima of the Na^+^–O_water and Na^+^–O_acetate RDFs remained at 2.33 and 2.03 Å, respectively, and the total first-shell coordination number was nearly unchanged (5.248 versus 5.259). The Na^+^–O_PAM coordination number was only 0.084, indicating that PAM provides sparse carbonyl contacts rather than acting as a dominant Na^+^ ligand (Figures S14–S15 and Table S2). MSD analysis showed that PAM reduced the apparent diffusion coefficients of acetate and water by 11.6% and 6.5%, respectively, whereas the small change in Na^+^ mobility was sensitive to the fitting window and was therefore not considered robust (Figure S16 and Table S3). These results support a kinetic-confinement mechanism in which PAM preserves the primary Na^+^–water/acetate coordination motifs while modestly restricting ion–solvent mobility, thereby potentially hindering the cooperative rearrangement required for spontaneous nucleation. Mechanical stimulation may locally perturb this metastable organization and facilitate nucleation, after which thermodynamically favorable crystallization proceeds. Because only one 1 ns production trajectory was analyzed for each model, these simulations characterize pre-nucleation coordination and mobility rather than directly reproducing the rare nucleation event or the macroscopic crystallization wavefront.

Note S4**:** Analysis of the morphological structure of SCG-MNs

The needle array adopts a 10 × 10 configuration with 500 μm base width and 1600 μm height (Figure S19). Scanning electron microscope (SEM) images of SCG-MNs patch reveal its structural integrity under a casting condition with ≈41.7% water content, which is contributed by the mechanical robustness endowed by its lattice structure (Figure S20a). The EDS analysis of the enlarged cross-section of a single needle body indicates the uniform distribution of all constituent elements, with no clusters or micron-scale elemental phase separation (Figure S20b). The orientation striations shown in SCGs’ polarized images reveal that the advancement of lattice layer generates micrometer-scale prisms perpendicular to nucleation points, attributable to the guiding effect of polyacrylamide network framework on crystal fronts (Figure 2c). To investigate the internal crystalline pillar morphology of SCG-MNs, samples were cryo-fractured in liquid nitrogen and vacuum-dehydrated for analysis. As shown in Figure S2, cross-sectional SEM images reveal highly oriented crystalline prisms with diameters of approximately 1.8 μm, providing further evidence of this guiding effect.

**Note S5:** Comparison of drug-delivery kinetics

Table S1 broadly compares the reported half-release times of SCG-MNs with representative drug-delivery platforms employing different materials, release mechanisms, and intended release profiles. This comparison is retained to contextualize the rapid intrinsic release of SCG-MNs rather than establish a universal performance ranking. To provide a more relevant benchmark, Figure S25 separately compares SCG-MNs with ten microneedle systems developed for rapid delivery.^[1–10]^ Because these studies reported different endpoints—including cumulative release, skin delivery or deposition, structural dissolution, and effective application—the original endpoint definitions were retained. Linear interpolation of the SCG-MN release profile yielded T_50_ ≈ 32 s, T_75_ ≈ 149 s, and T_80_ ≈ 207 s. The latter thresholds enable percentage-matched comparisons with the values reported by Sheng et al. (>75% at approximately 120 s) and Than et al. (>80% at 300 s in simulated tear fluid).^[4,8]^ Overall, SCG-MNs fall within the minute-scale rapid-delivery range, although these heterogeneous endpoints should not be interpreted as directly interchangeable kinetic parameters.

**Note S6:** Application-window leachable exposure of SCG-MNs

To relate the chemical leaching results to the cytocompatibility dataset, NaAc-derived Na+ and residual acrylamide (AAm) were quantified by ion chromatography (IC) and high-performance liquid chromatography (HPLC), respectively, after extracting SCGs at an SCG-to-water ratio of 1:20 for 10 min or 24 h (Figures S27 and S28). The 24 h condition, consistent with the extraction duration used in the L929 cell assays, yielded 6.940 mg mL^−1^ Na^+^ and 166.174 μg mL^−1^ AAm. At the application-relevant 10 min point, these concentrations were 1.560 mg mL^−1^ and 14.716 μg mL^−1^, corresponding to 22.5% and 8.9% of their respective 24-h values. Using the ≈5 wt% 24-h extract as the reference, the apparent equivalent SCG loadings were calculated as C_eq_ = 5 wt% × C_10 min_/C_24 h_, giving approximately 1.12 wt% for Na^+^ and 0.44 wt% for AAm. These levels were comparable to or below the 1 wt% condition that maintained L929 cytocompatibility. Together with the 89.33% cumulative drug release achieved within 10 min, these results show that the rapid-delivery window combines effective payload release with leachable concentrations within the low-concentration cytocompatible range.

Figure S1**.** Schematic illustrating the effects of temperature, excitation force, sodium acetate concentration, system acidity, and relative humidity on transition dynamics of supercooled crystallization.

Figure S2**.** The tomographic SEM image of the SCGs after vacuum freezing to remove the liquid, as well as the analysis of the diameter of the lattice beam and the local magnified image. Scale bars, 15 μm, 2 μm.

Figure S3. XRD patterns of SCGs and its grain sizes calculated using the Xerxes formula.

Figure S4. Crystal-front layer growth rate of SCGs under a wide range of epidermal contact forces.

**Figure S5.** Packaging and short-distance transportation stability of metastable SCGs. (i) Packaging procedure, including individual storage in rigid glass vials, foam cushioning, and carton packaging. (ii) Representative appearance of ten SCG samples and a crystallized control after approximately 30 km of transportation. (iii) Paired normalized grayscale intensities before and after transportation. The ROI intensity of each sample was normalized to that of the crystallized control acquired under the same imaging condition. Gray lines connect measurements from the same vial; Error bars represent mean ± SD (n = 10 independent vials).

**Figure S6.** Crystal-front velocity of SCGs at different temperatures. Data are mean ± SD (n = 3).

**Figure S7.** Composition-dependent mechanical reliability and thermal lattice disassembly of SCGs. a) Compressive stress–strain curves of crystallized SCGs prepared at NaAc : total polymerizable monomer : water feed mass ratios of 6:1:5 and 6:0.5:5. Three independently prepared 6:0.5:5 specimens are shown. Two of the three specimens exhibited premature mechanical instability at approximately 2% strain, indicating limited mechanical reproducibility at the lower polymer content. The inset enlarges the low-strain region. b) Representative appearance of crystallized SCGs after sealed heating at 85 °C. The 6:1:5 formulation underwent complete lattice disassembly and became transparent, whereas the 6:2:5 specimens retained opaque crystalline regions, demonstrating incomplete thermal lattice disassembly at the higher polymer content.

**Figure S8**. Effect of cation substitution on phase switching and mechanical behavior. (i, ii) NaAc-based SCGs and Ca^2+^- or Mg^2+^-substituted formulations after cooling to room temperature and subsequent mechanical stimulation. (iii–v) Qualitative load-bearing, deformation, and post-loading shape-recovery behavior of the three formulations.

**Figure S9.** Temperature-dependent thermal lattice-disassembly kinetics of SCGs. a) Thermal disassembly fraction at 55–85 °C. b,c) Corresponding t_50_ and t_90_ values. Data are mean ± SD (n = 3).

**Figure S10.** Water-assisted radial lattice-disassembly kinetics of SCGs. **a)** Schematic of water exposure, time-lapse imaging, and crystalline-area segmentation. **b)** Residual crystalline-area fraction. **c)** Equivalent radial retreat; the dashed line denotes linear fitting. Data are mean ± SD (n = 3).

Figure S11**.** Temperature-variable FTIR spectra of the SCGs upon heating from 30 to 150 °C in the range of **a)** 3800–3000 and **b)** 1625-1500 cm^–1^. PCMW2D synchronous spectra of SCGs upon heating from 30 to 150 °C in the range of **c)** 3800–3000 and **d)** 1625-1500 cm^-1^.

Figure S12**.** Molecular dynamics simulation snapshot of lattice formation processes. **a)** shows the initial start-up, while **b)** shows the stability within the box after it has been activated.

Figure S13**.** Simulated calculations of the mean square displacement (MSD) and self-diffusion coefficient of Na ions.

**Figure S14.** Representative equilibrated configurations of (i) NaAc–H_2_O and (ii) PAM–NaAc shown using the same view and colour scheme; PAM chains are shown in magenta.

**Figure S15.** Local coordination and molecular mobility in NaAc–H_2_O and PAM–NaAc. a,b), Na^+^ RDFs with (a) water oxygen and (b) acetate oxygen. c), Na^+^–PAM carbonyl-oxygen RDF; the circle and dashed line mark the first maximum and minimum. d), Na^+^ first-shell coordination composition. e), PAM/control apparent diffusion-coefficient ratios from the 100–500 ps fit; the Na^+^ bar denotes fitting-window sensitivity.

**Figure S16.** MSD analysis and fitting-window sensitivity. a–c), MSDs of (a) acetate, (b) water, and (c) Na^+^; shading marks the nominal 100–500 ps fit. d), PAM/control apparent diffusion-coefficient ratios across fitting windows.

Figure S17**.** The dehydration curves of SCGs with a lattice structure and the same weight and liquid content as the polyacrylamide gel without lattice structure. Error bars represent SD (n = 3)

**Figure S18.** Mechanical performance of SCGs after repeated recrystallization cycles. a) Compressive stress–strain curves of the initial SCGs and samples after 1, 3, and 10 cycles. b) Corresponding compressive moduli. Each cycle comprised thermal lattice disassembly, cooling, and mechanically triggered recrystallization. Error bars represent SD (n = 3)

Figure S19**.** Optical images of the SCG-MNs patch captured using an optical microscope. Scale bars, 2 mm.

Figure S20**.** **a)** The SEM images reveal the complete morphology of the needle bodies at different scales of SCG-MNs. Among them, (iv) shows the morphology after dehydration of the needle base surface. Scale bars, (i) 500 μm, (ii) 200 μm, (iii) 200 μm, (iv) 100 μm. **b)** The element mapping of image (iv) reveals the uniformity of SCG-MNs. Scale bar, 100 μm.

**Figure S21.** Mechanical performance of rigid-state SCG-MNs after long-term storage. a) Representative compressive force–displacement curves of freshly prepared and approximately 480-day-stored SCG-MNs. The aged sample showed higher initial stiffness but reduced fracture displacement and force. b) Corresponding fracture force per needle, which decreased from 2.28 to 1.80 N needle^-1^ (78.8% of the initial value) but remained above the epidermal-penetration requirement of 0.05 N needle⁻¹

**Figure S22.** Dehydration-induced rehardening of SCG-MNs after skin application. (i) SCG-MN patch after water-assisted softening and subsequent dehydration. (ii, iii) Load-bearing demonstration of the re-hardened patch using a 1 kg weight. (iv) Patch after unloading.

**Figure S23.** Structural retention of SCG-MNs during skin application and removal. (i) SCG-MN patch before insertion. (ii) Application to ex vivo porcine skin. (iii) Corresponding puncture sites. (iv) Retrieved softened patch showing retained needle profiles.

**Figure S24.** Optical assessment of SCG-MN integrity following ex vivo skin application and removal. a–c) Binary maps of three independent 10 × 10 patches. Each square represents one microneedle; gray squares indicate intact needles and orange crossed squares indicate visibly damaged needles. d) Patch-level needle integrity. The dashed line and shaded region represent the mean and standard deviation, respectively. Overall, 292 of 300 needles retained intact optical profiles (97.3 ± 0.6%; n = 3 independent patches).

**Figure S25.** Comparison of SCG-MNs with microneedle platforms specifically developed for rapid drug delivery. a) Time-dependent cumulative release or delivery efficiencies reported for representative rapid-delivery microneedle systems. The complete SCG-MN release profile is shown, whereas literature values are presented only at their originally reported time points. b) Endpoint-aware timeline summarizing ten rapid-delivery microneedle studies. Marker shapes distinguish cumulative release, skin deposition, structural dissolution, and effective application endpoints.

Figure S26**.** a) Cell toxicity evaluation of L929 cells after treatment with SCG-MNs for 1, 3, and 5 days. b) TRITC/DAPI staining of L929 cells after exposed to 1 wt% and 5 wt% SCG-MNs for 1 day. Scale bar, 100 µm.

**Figure S27.** HPLC quantification of residual acrylamide released from SCGs. a), Baseline-corrected and peak-normalized chromatograms of the blank, AAm standard, and SCG extracts collected at 10 min and 24 h. b), AAm calibration curve over 10–200 μg mL^-1^; diamonds indicate the measured samples. c), AAm concentrations and corresponding mass-normalized release from SCGs.

**Figure S28.** IC quantification of sodium-ion release from SCGs. a), Baseline-corrected and peak-normalized ion chromatograms of SCG extracts collected at 10 min and 24 h. b), Na⁺ concentrations after correction for the 10- and 50-fold sample dilutions, together with the corresponding mass-normalized release from SCGs.

**Figure S29.** Transient thermal response of SCG-MNs during skin application. Infrared thermographic images show that the surface temperature peaked at 44.4 °C after triggering and subsequently decreased to 29.7 °C within 20 min. Because thermal injury depends on both temperature and exposure duration, this brief temperature elevation indicates a low risk of thermal damage under the tested conditions.

Table S1**.** Comparison of the half release time of drug for SCG-MNs patch and other recently reported advanced drug delivery devices.

| **Material type** | **Delivery vector** | **Preparation method** | **Half release time (s)** | **References** |
| --- | --- | --- | --- | --- |
| Effervescent agent | Microneedle | In situ photopolymerization | 2448s | ^[11]^ |
| *N*-vinylpyrrolidone | Microneedle | In situ photopolymerization | 52s | ^[4]^ |
| Glucose-responsive | Microneedle | In situ photopolymerization | 3600s | ^[12]^ |
| Self-Oxygenating | Microneedle | Centrifugation-assisted stepwise moulding | 1285s | ^[13]^ |
| PLA | Microneedle | Concentration-solidification | 1728000s | ^[14]^ |
| Poly(ionic liquid) | Microneedle | In situ photopolymerization | 10332s | ^[15]^ |
| Hollow biocompatible resin | Microneedle | 3D-Printing | 354s | ^[16]^ |
| Dissolving-type | Microneedle | Vacuum-assisted lyophilization | 4326s | ^[17]^ |
| Catechol-based | Microneedle | Self-crosslinking-curing | 1686s | ^[18]^ |
| Photodynamic gel-bombs | Drug-loaded microstructure | Ion-crosslinked suspension gelation | 41616s | ^[19]^ |
| Biomineral | Drug-loaded microstructure | Compression mineralization | 164160s | ^[20]^ |
| Oil gel | Drug-loaded microstructure | Melt gelation | 924s | ^[21]^ |
| Gel-microspheres | Drug-loaded microstructure | Microfluidic technology | 169344s | ^[22]^ |
| Mesoporous NPs | Drug-loaded microstructure | Micelle template-assisted reductive precipitation | 27792s | ^[23]^ |
| Zwitterion gel | Drug-loaded microstructure | Microfluidic technology | 406944s | ^[24]^ |
| Janus | Hydrogel patch | In situ synchronous dual crosslinking | 59616s | ^[25]^ |
| Gelatin | Hydrogel patch | Benzyl crosslinking | 777600s | ^[26]^ |
| Shear-Thinning | Hydrogel patch | Solution gelation | 211680s | ^[27]^ |
| Polysaccharide | Hydrogel patch | In situ photopolymerization | 228960s | ^[28]^ |
| Brushpolymer | Hydrogel patch | Supramolecular cross-linking gelation | 14652s | ^[29]^ |
| Recomb Protein | Hydrogel patch | Supramolecular cross-linking gelation | 20808s | ^[30]^ |
| **This work** | **Microneedle** | **In situ photopolymerization** | **~30s** | **This work** |

**Table S2.** First-shell RDF parameters and coordination numbers. Each coordination number was obtained by integrating the corresponding RDF to its own first minimum

| **System** | **O pair** | **r_peak (Å)** | **r_min (Å)** | **Coordination number** |
| --- | --- | --- | --- | --- |
| NaAc–H₂O | O_acetate | 2.03 | 2.87 | 2.882 |
| NaAc–H₂O | O_water | 2.33 | 3.13 | 2.366 |
| PAM–NaAc | O_acetate | 2.03 | 2.93 | 2.871 |
| PAM–NaAc | O_water | 2.33 | 3.05 | 2.304 |
| PAM–NaAc | O_PAM | 2.30 | 3.30 | 0.084 |

**Table S3.** Apparent diffusion coefficients obtained from the 100–500 ps MSD fits.

| **Species** | **NaAc–H₂O D (Å^2^ ps^-1^)** | **PAM–NaAc D (Å^2^ ps^-1^)** | **Change** | **R^2^: control / PAM** |
| --- | --- | --- | --- | --- |
| Acetate | 7.034 × 10^-3^ | 6.216 × 10^-3^ | −11.6% | 0.9987 / 0.9996 |
| Water | 3.595 × 10^-2^ | 3.360 × 10^-2^ | −6.5% | 1.0000 / 0.9998 |
| Na^+^ | 7.414 × 10^-3^ | 7.731 × 10^-3^ | +4.3% | 0.9995 / 0.9997 |

**Table S4.** Complete source records used for the quantitative curve and endpoint-aware timeline in Figure S25.

| **Panel** | **Study** | **Series or endpoint label** | **Time (s)** | **Value (%)** | **Metric or endpoint type** | **Lower-bound flag** | **References** |
| --- | --- | --- | --- | --- | --- | --- | --- |
| a | SCG-MNs | SCG-MNs | 10 | 37.76 | cumulative release | No | This work |
| a | SCG-MNs | SCG-MNs | 20 | 41.88 | cumulative release | No | This work |
| a | SCG-MNs | SCG-MNs | 30 | 49.19 | cumulative release | No | This work |
| a | SCG-MNs | SCG-MNs | 60 | 62.02 | cumulative release | No | This work |
| a | SCG-MNs | SCG-MNs | 120 | 72.44 | cumulative release | No | This work |
| a | SCG-MNs | SCG-MNs | 300 | 88.12 | cumulative release | No | This work |
| a | SCG-MNs | SCG-MNs | 600 | 89.33 | cumulative release | No | This work |
| a | Baek et al. (2017) | Baek (skin delivery) | 60 | 69 | skin delivery efficiency | No | [6] |
| a | Baek et al. (2017) | Baek (skin delivery) | 120 | 77 | skin delivery efficiency | No | [6] |
| a | Baek et al. (2017) | Baek (skin delivery) | 300 | 84 | skin delivery efficiency | No | [6] |
| a | Sheng et al. (2023) | Sheng | 120 | >75 | cumulative release | Yes | [4] |
| a | Than et al. (2018) | Than (simulated tear) | 300 | >80 | cumulative release | Yes | [8] |
| a | Than et al. (2018) | Than (gelatin hydrogel) | 1800 | >80 | cumulative release | Yes | [8] |
| b | SCG-MNs | T_50_ = 32 s | 31.9 | — | cumulative release | n.a. | This work |
| b | SCG-MNs | T_75_ = 149 s | 149.4 | — | cumulative release | n.a. | This work |
| b | SCG-MNs | T_80_ = 207 s | 206.8 | — | cumulative release | n.a. | This work |
| b | Gill & Prausnitz (2007) | Coating dissolution | 20 | — | structural dissolution | n.a. | [1] |
| b | Zhang et al. (2012) | Therapeutic tissue level | 60 | — | effective application | n.a. | [2] |
| b | Zheng et al. (2025) | Complete tip dissolution | 90 | — | structural dissolution | n.a. | [3] |
| b | Sheng et al. (2023) | >75% release | 120 | — | cumulative release | Shown in label | [4] |
| b | Zhu et al. (2014) | Needle dissolution/release | 120 | — | structural dissolution | n.a. | [5] |
| b | Baek et al. (2017) | Coating release in PBS | 120 | — | skin delivery | n.a. | [6] |
| b | You et al. (2023) | Effective administration | 300 | — | effective application | n.a. | [7] |
| b | Than et al. (2018) | >80% release in simulated tear | 300 | — | cumulative release | Shown in label | [8] |
| b | Cormier et al. (2004) | Pharmacologically relevant delivery | 300 | — | effective application | n.a. | [9] |
| b | Sedky et al. (2024) | Ex vivo deposition window | 300 | — | skin delivery | n.a. | [10] |

References

[1]	Gill, H. S., Prausnitz, M. R., "Coated microneedles for transdermal delivery," *Journal of Controlled Release* 117, no. 2 (2007), <https://doi.org/10.1016/j.jconrel.2006.10.017>.

[2]	Zhang, Y., Brown, K., Siebenaler, K., et al., "Development of Lidocaine-Coated Microneedle Product for Rapid, Safe, and Prolonged Local Analgesic Action," *Pharmaceutical Research* 29, no. 1 (2012), <https://doi.org/10.1007/s11095-011-0524-4>.

[3]	Zheng, H., Liu, N., Zhou, T., et al., "Rapidly dissolving and detachable microneedles loaded with antimicrobial peptides for acne vulgaris treatment," *Journal of Materials Chemistry B* 13, no. 46 (2025), <https://doi.org/10.1039/d5tb00845j>.

[4]	Sheng, T., Jin, R., Yang, C., et al., "Unmanned Aerial Vehicle Mediated Drug Delivery for First Aid," *Advanced Materials* 35, no. 10 (2023), <https://doi.org/10.1002/adma.202208648>.

[5]	Zhu, Z., Luo, H., Lu, W., et al., "Rapidly Dissolvable Microneedle Patches for Transdermal Delivery of Exenatide," *Pharmaceutical Research* 31, no. 12 (2014), <https://doi.org/10.1007/s11095-014-1424-1>.

[6]	Baek, S. H., Shin, J. H., Kim, Y. C., "Drug-coated microneedles for rapid and painless local anesthesia," *Biomedical Microdevices* 19, no. 1 (2017), <https://doi.org/10.1007/s10544-016-0144-1>.

[7]	You, J., Yang, C., Han, J., et al., "Ultrarapid-Acting Microneedles for Immediate Delivery of Biotherapeutics," *Advanced Materials* 35, no. 45 (2023), <https://doi.org/10.1002/adma.202304582>.

[8]	Than, A., Liu, C., Chang, H., et al., "Self-implantable double-layered micro-drug-reservoirs for efficient and controlled ocular drug delivery," *Nature Communications* 9, no. 1 (2018), <https://doi.org/10.1038/s41467-018-06981-w>.

[9]	Cormier, M., Johnson, B., Ameri, M., et al., "Transdermal delivery of desmopressin using a coated microneedle array patch system," *Journal of Controlled Release* 97, no. 3 (2004), <https://doi.org/10.1016/j.jconrel.2004.04.003>.

[10]	Sedky, M., Ali, A., Abdel-Mottaleb, M., et al., "A new rapid-release SMA-activated micropump with incorporated microneedle arrays and polymeric nanoparticles for optimized transdermal drug delivery," *Sensors and Actuators B: Chemical* 408 (2024), <https://doi.org/10.1016/j.snb.2024.135549>.

[11]	Han, J., Wu, Z., Zhan, S., et al., "Biorhythm-mimicking growth hormone patch," *Nature Materials* (2025), <https://doi.org/10.1038/s41563-025-02188-9>.

[12]	Yu, J., Wang, J., Zhang, Y., et al., "Glucose-responsive insulin patch for the regulation of blood glucose in mice and minipigs," *Nature Biomedical Engineering* 4, no. 5 (2020), <https://doi.org/10.1038/s41551-019-0508-y>.

[13]	Jiang, X., Lai, Y., Xia, W., et al., "Self-Oxygenating PROTAC Microneedle for Spatiotemporally-Confined Protein Degradation and Enhanced Glioblastoma Therapy," *Advanced Materials* 37, no. 13 (2025), [https://doi.org/10.1002/adma.202411869](https://doi.org/https://doi.org/10.1002/adma.202411869).

[14]	Li, W., Terry, R. N., Tang, J., Feng, M. R., Schwendeman, S. P., Prausnitz, M. R., "Rapidly separable microneedle patch for the sustained release of a contraceptive," *Nature Biomedical Engineering* 3, no. 3 (2019), <https://doi.org/10.1038/s41551-018-0337-4>.

[15]	Gao, S., Rao, Y., Wang, X., et al., "Chlorella-Loaded Antibacterial Microneedles for Microacupuncture Oxygen Therapy of Diabetic Bacterial Infected Wounds," *Advanced Materials* 36, no. 15 (2024), [https://doi.org/10.1002/adma.202307585](https://doi.org/https://doi.org/10.1002/adma.202307585).

[16]	Rajesh, N. U., Hwang, J., Xu, Y., et al., "3D-Printed Latticed Microneedle Array Patches for Tunable and Versatile Intradermal Delivery," *Advanced Materials* 36, no. 44 (2024), [https://doi.org/10.1002/adma.202404606](https://doi.org/https://doi.org/10.1002/adma.202404606).

[17]	Wang, H., Cai, R., Wang, S., et al., "A wearable transdermal device for on-demand drug delivery," *Matter* 8, no. 4 (2025), [https://doi.org/10.1016/j.matt.2025.102040](https://doi.org/https://doi.org/10.1016/j.matt.2025.102040).

[18]	GhavamiNejad, A., Liu, J. F., Mirzaie, S., et al., "Catechol-based chemistry for hypoglycemia-responsive delivery of zinc-glucagon via hydrogel-based microneedle patch technology," *Nature Communications* 16, no. 1 (2025), <https://doi.org/10.1038/s41467-025-58278-4>.

[19]	Bai, X., Meng, F., Wang, X., et al., "Photodynamic gel-bombs enhance tumor penetration and downstream synergistic therapies," *Signal Transduction and Targeted Therapy* 10, no. 1 (2025), <https://doi.org/10.1038/s41392-025-02186-y>.

[20]	Han, J., Wang, S., Fang, W., et al., "Long-acting IL-2 release from pressure-fused biomineral tablets promotes antitumor immune response," *Nature Cancer* 6, no. 8 (2025), <https://doi.org/10.1038/s43018-025-00993-4>.

[21]	Kirtane, A. R., Karavasili, C., Wahane, A., et al., "Development of oil-based gels as versatile drug delivery systems for pediatric applications," *Science Advances* 8, no. 21, <https://doi.org/10.1126/sciadv.abm8478>.

[22]	Li, W., Chen, J., Zhao, S., et al., "High drug-loaded microspheres enabled by controlled in-droplet precipitation promote functional recovery after spinal cord injury," *Nature Communications* 13, no. 1 (2022), <https://doi.org/10.1038/s41467-022-28787-7>.

[23]	Li, Z., Yang, J., Ren, B., et al., "Double-Layered Hollow Mesoporous Cuprous Oxide Nanoparticles for Double Drug Sequential Therapy of Tumors," *Advanced Materials* 36, no. 28 (2024), [https://doi.org/10.1002/adma.202313212](https://doi.org/https://doi.org/10.1002/adma.202313212).

[24]	Hou, J., Lin, Y., Zhu, C., et al., "Zwitterion-Lubricated Hydrogel Microspheres Encapsulated with Metformin Ameliorate Age-Associated Osteoarthritis," *Advanced Science* 11, no. 30 (2024), [https://doi.org/10.1002/advs.202402477](https://doi.org/https://doi.org/10.1002/advs.202402477).

[25]	Freedman, B. R., Kuttler, A., Beckmann, N., et al., "Enhanced tendon healing by a tough hydrogel with an adhesive side and high drug-loading capacity," *Nature Biomedical Engineering* 6, no. 10 (2022), <https://doi.org/10.1038/s41551-021-00810-0>.

[26]	Huang, Y., Chen, T., Ren, C., et al., "High-Strength Gelatin Hydrogel Scaffold with Drug Loading Remodels the Inflammatory Microenvironment to Enhance Osteoporotic Bone Repair," *Advanced Materials* 37, no. 13 (2025), [https://doi.org/10.1002/adma.202501051](https://doi.org/https://doi.org/10.1002/adma.202501051).

[27]	Zhang, Y., Miao, D., Su, M., et al., "Synergistic Drug-Loaded Shear-Thinning Star Polymer Hydrogel Facilitates Gastrointestinal Lesion Resection and Promotes Wound Healing," *Advanced Science* 11, no. 26 (2024), [https://doi.org/10.1002/advs.202309586](https://doi.org/https://doi.org/10.1002/advs.202309586).

[28]	Zhao, M., Xiang, J., Meng, Y., et al., "Astragalus Polysaccharide Hydrogels with Drug-Carrying Super Self-Assembly from Natural Herbs Promote Wound Healing," *ACS Nano* 19, no. 23 (2025), <https://doi.org/10.1021/acsnano.5c03744>.

[29]	Shukla, A., Singh, A. P., Maiti, P., "Injectable hydrogels of newly designed brush biopolymers as sustained drug-delivery vehicle for melanoma treatment," *Signal Transduction and Targeted Therapy* 6, no. 1 (2021), <https://doi.org/10.1038/s41392-020-00431-0>.

[30]	Jiang, X., Feng, T., An, B., et al., "A Bi-Layer Hydrogel Cardiac Patch Made of Recombinant Functional Proteins," *Advanced Materials* 34, no. 19 (2022), [https://doi.org/10.1002/adma.202201411](https://doi.org/https://doi.org/10.1002/adma.202201411).
